# Designing a multi-epitope vaccine candidate to combat MERS-CoV by employing an immunoinformatics approach

**DOI:** 10.1038/s41598-021-92176-1

**Published:** 2021-07-29

**Authors:** Shafi Mahmud, Md. Oliullah Rafi, Gobindo Kumar Paul, Maria Meha Promi, Mst. Sharmin Sultana Shimu, Suvro Biswas, Talha Bin Emran, Kuldeep Dhama, Salem A. Alyami, Mohammad Ali Moni, Md. Abu Saleh

**Affiliations:** 1 Microbiology Laboratory, Department of Genetic Engineering and Biotechnology, University of Rajshahi, Rajshahi, 6505 Bangladesh; 2 Department of Genetic Engineering and Biotechnology, Jashore University of Science and Technology, Jashore, 7408 Bangladesh; 3 Department of Genetic Engineering and Biotechnology, University of Rajshahi, Rajshahi, 6505 Bangladesh; 4 Department of Pharmacy, BGC Trust University Bangladesh, Chittagong, 4381 Bangladesh; 5 Division of Pathology, ICAR-Indian Veterinary Research Institute, Izatnagar, Bareilly, 243122 Uttar Pradesh India; 6 Faculty of Medicine, WHO Collaborating Centre on eHealth, UNSW Digital Health, School of Public Health and Community Medicine, UNSW Sydney, Sydney, NSW 2052 Australia; 7 Department of Mathematics and Statistics, Imam Mohammad Ibn Saud Islamic University, Riyadh, 11432 Saudi Arabia

**Keywords:** Computational biology and bioinformatics, Drug discovery

## Abstract

Currently, no approved vaccine is available against the Middle East respiratory syndrome coronavirus (MERS-CoV), which causes severe respiratory disease. The spike glycoprotein is typically considered a suitable target for MERS-CoV vaccine candidates. A computational strategy can be used to design an antigenic vaccine against a pathogen. Therefore, we used immunoinformatics and computational approaches to design a multi-epitope vaccine that targets the spike glycoprotein of MERS-CoV. After using numerous immunoinformatics tools and applying several immune filters, a poly-epitope vaccine was constructed comprising cytotoxic T-cell lymphocyte (CTL)-, helper T-cell lymphocyte (HTL)-, and interferon-gamma (IFN-γ)-inducing epitopes. In addition, various physicochemical, allergenic, and antigenic profiles were evaluated to confirm the immunogenicity and safety of the vaccine. Molecular interactions, binding affinities, and the thermodynamic stability of the vaccine were examined through molecular docking and dynamic simulation approaches, during which we identified a stable and strong interaction with Toll-like receptors (TLRs). In silico immune simulations were performed to assess the immune-response triggering capabilities of the vaccine. This computational analysis suggested that the proposed vaccine candidate would be structurally stable and capable of generating an effective immune response to combat viral infections; however, experimental evaluations remain necessary to verify the exact safety and immunogenicity profile of this vaccine.

## Introduction

Middle East respiratory syndrome coronavirus (MERS-CoV) originated in bats and causes an acute, infectious, viral respiratory disease characterized by symptoms including cough, fever, diarrhea, and occasional multi-organ failure^1,2^. MERS-CoV is covered by a single-stranded RNA and belongs to the Beta-coronavirus (β-CoV) genus in the family of Coronaviridae, which is distinct from the coronaviruses that cause the common cold coronavirus and severe acute respiratory syndrome (SARS)^3–5^. MERS-CoV was first identified in Saudi Arabia in 2012 and later spread to European countries. MERS-CoV is associated with an unusually high mortality rate of approximately 35%^6–8^. According to the WHO report released in May 2018, a total of 2,220 people contracted MERS-CoV, including 790 deaths, resulting in a mortality rate of 35.6%. In Saudi Arabia alone, 1,844 cases were identified, resulting in 716 deaths. In 2015, the South Korean population also became infected with this acute virus, resulting in 186 cases and 36 deaths^5,9^.

The genome for MERS-CoV encodes envelope (E), membrane (M), nucleocapsid (N), and spike (S) structural proteins, which are necessary to complete the structure of this viral particle^10,11^. The E protein is typically expressed when cells become infected and is distributed within the intracellular membranes of the endoplasmic reticulum (ER) and Golgi compartments^12–14^. The N protein binds to the genome of the CoV-RNA, forming the nucleocapsid^15^, whereas the M protein defines the shape of the viral envelope^16^. The spike (S) glycoprotein is another important protein because it is involved in host recognition and facilitates host cell entry^17,18^. The spike protein consists of S1 and S2 subunits^19,20^. To initiate infection, the S1 subunit binds to the dipeptidyl peptidase 4 (DPP4) receptor on the host cell surface^19,20,21^, and the S2 subunit mediates the fusion of the viral and host cell membranes^20^. As of 2020, no specific vaccines against MERS-CoV are available. The S glycoprotein has been considered to be a potential vaccine candidate because neutralizing antibodies against this protein would block viral entry and prevent viral infection^23–25^.

Using conventional methods, vaccines are designed using large proteins, and the use of inappropriate antigens can increase the potential for allergic reactions; however, a peptide-based multi-epitope vaccine that contains short antigenic peptide fragments, referred to as epitopes, might be able to overcome these limitations^26^. Epitopes, the antigenic portion of the pathogen that is recognized by the host immune system, and innate immunity are elicited against it^27^. The cell-mediated immune response is mainly dependent on the pattern recognition receptors recognizing the pathogen-associated molecular patterns of the pathogen^28^. Toll-like receptors are considered pathogen recognition receptors (PRRs), TLRs family consists of eleven proteins, and each of them uniquely interacts with diverse PAMPs, they are expressed on the surface of the cells^29^. Toll-like receptor-2 can recognize viral structural glycoproteins. The TLR4 has an essential role during host pathogenesis that can trigger the anti-viral host defense mechanisms against coronavirus^30^. After infection, cytotoxic T-cell lymphocytes (CTLs) become activated and kill infected cells^31^. Antigens bound to the major histocompatibility complex (MHC) are presented on the infected cell surface, allowing them to be recognized by CTLs^32^. MHC class I molecules exhibit cytosolic peptides antigens of the infected cells and phagocytosed antigens are presented on MHC class II molecules^33–36^. CD4 + T cells recognize the antigenic peptides that are displayed by class II MHC molecules whereas cytotoxic T-cell lymphocyte (CTL)-interact with class I MHC-peptide complexes^37^. Activated CD4 + T cells secrete cytokines and are responsible for the further activation of B cells required for producing proper antibodies^38^.

**Figure 1:**
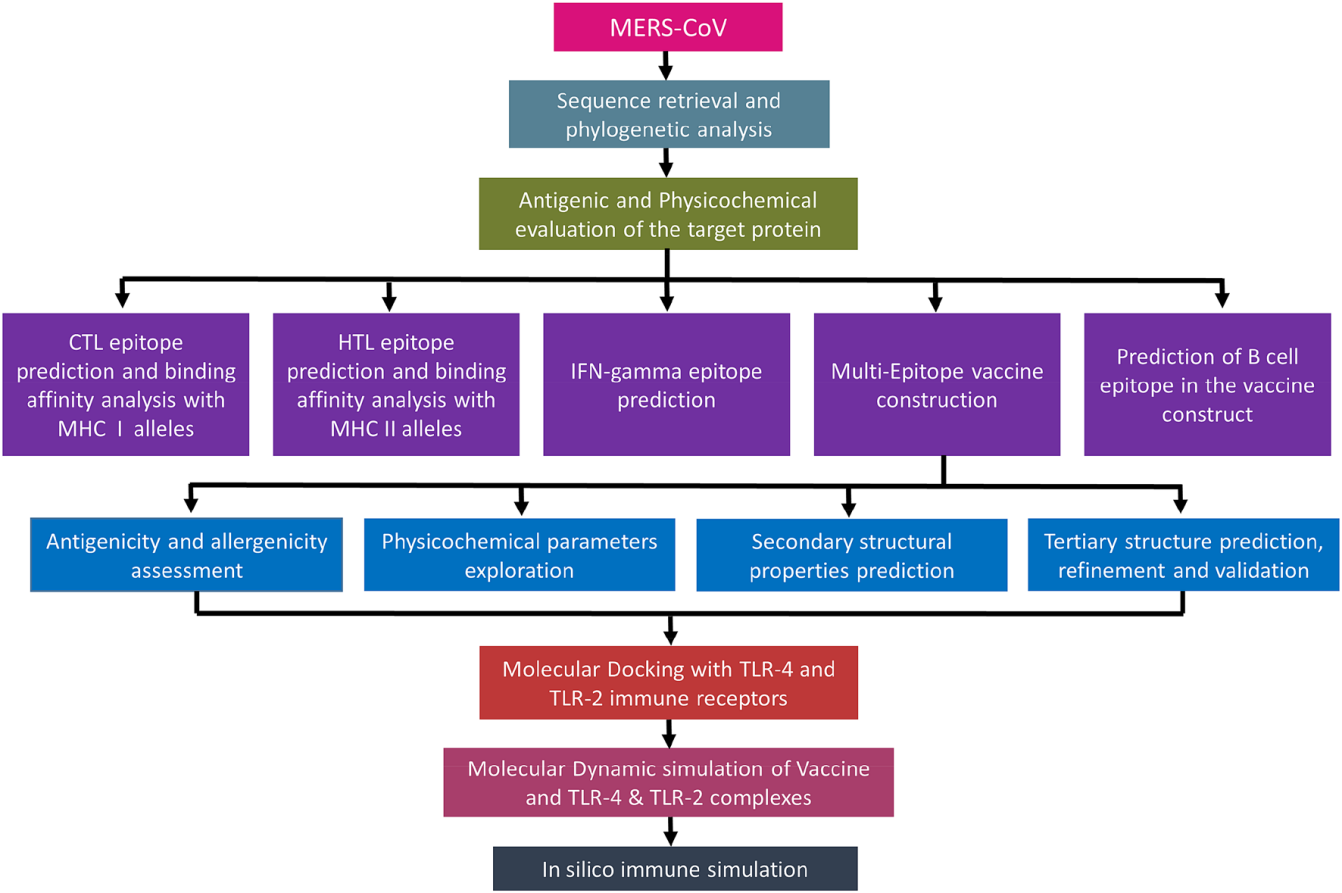
Graphical representation of the overall systematic study which shown: selection sequence (antigenic proteins) that suitable for phylogenetic analysis, epitope prediction from the goal protein, construction of vaccine, prediction of B cell epitope, feature assessment of target vaccine. Molecular docking of the vaccine with TLR-2 and TLR-4 immune receptor. Molecular dynamic simulations to estimate the stability of docked complexes. Finally, immune stimulation was employed to understand the immune efficiency of the target vaccine.

In this study, an immunoinformatics strategy was applied in a sequential manner to design a multi-epitope subunit vaccine to target the MERS-CoV S glycoprotein. This epitope-based vaccine prediction was designed to be antigenic and non-allergenic in nature. The final vaccine contained cytotoxic T-cell lymphocyte (CTL)-, helper T-cell lymphocyte (HTL)-, and interferon-γ (IFN-γ)-inducing and B-cell epitope sequences. Additional physicochemical, molecular docking and thermodynamic stability profiling were assessed to evaluate the safety and efficiency of the vaccine (Fig. 1).

## Results

### Sequences retrieval and analysis of phylogenetic tree

**Figure 2:**
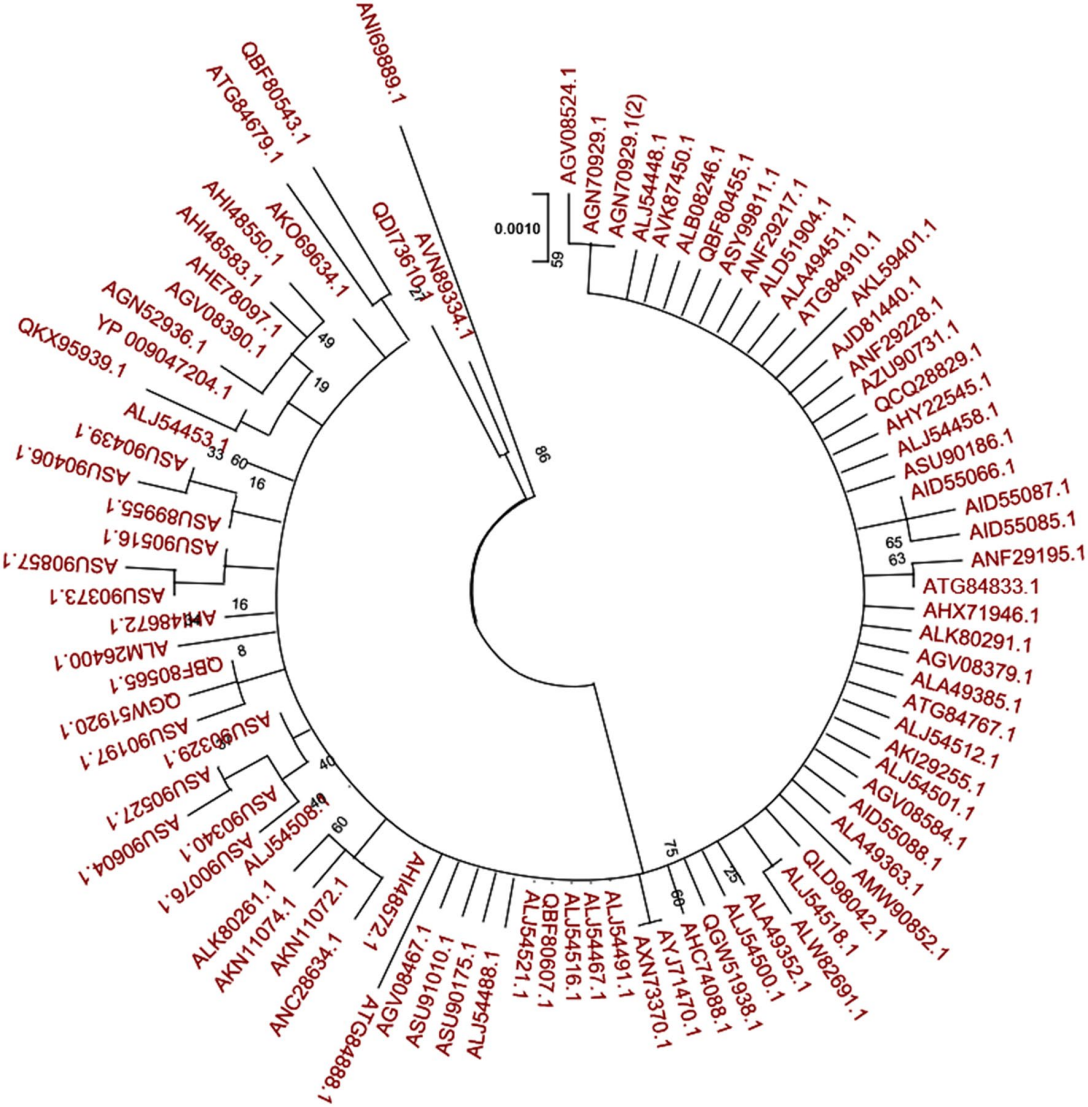
MERS-CoV spike glycoprotein that was taken from 11 different countries (Saudi Arabia, Lebanon, Yemen, Qatar, Egypt, Oman, Iran, United Arab Emirates, Jordan, England, and South Korea) and their phylogenetic analysis. The phylogenetic tree was constructed using the Maximum likelihood method with 1000 bootstrap replicates.

A phylogenetic tree was built using all of the spike glycoprotein sequences obtained from various MERS-CoV isolates identified in different countries (Fig. 2). The results showed that all of the S proteins assembled together into a single clade and were closely related to one another. Therefore, a MERS-CoV vaccine that was designed against one single strain could potentially be effective against all of the strains.

### Antigenic and physicochemical evaluations of the target protein

The VaxiJen v2.0 predicted score was 0.4919, which signified that the sequence was potently antigenic. The ExPASy tool in ProtParam indicated that the virulent target protein being used in this study has an instability index score of 36.53, an aliphatic index score of 83.00, and a negative grand average of hydropathicity (GRAVY) value of − 0.064 (Supplementary Table 1). The targeted spike glycoprotein contains 1353 amino acids with the molecular weight of 149,505.26 kDa which specifies the sequences to be antigenic. The estimated half-life was computed to be 30 h for mammalian-reticulocytes, > 20 h for yeast, > 10 h for *Escherichia coli*.

### The prediction of T-cell epitopes and assessment

The CTL epitopes can be used to eliminate virus-infected cells, generate cellular immunity, and reduce the level of circulating virus. In contrast, HTL epitopes can develop both cellular and humoral immune responses and have the ability to activate B-cells to produce antibodies^39^. Therefore, an effective vaccine should contain receptor-specific cytotoxic T-cell lymphocyte, helper T-cell lymphocyte epitopes. In this study, the NetCTL-1.2 server and the Infectious Disease Epidemiology Bureau (IDEB) stabilized matrix method (SMM) were employed for the prediction of the CTL epitope, whereas the HTL epitope was predicted using the Net MHCII pan 3.2 server (Supplementary Tables 2 and 3). In addition, the predicted sequences were subjected to numerous immune filters, including antigenicity, high-binding affinity, non-allergenicity, and promiscuously towards MHC (MHC-I and MHC-II) alleles. Finally, the best candidate was selected, as shown in Tables 1, 2, and Supplementary Table 4.

**Table 1:** Selected promiscuous cytotoxic T-lymphocyte epitope.

Epitopes	Position	HLA class 1 supertypes (Combined score)	MHC Class 1 alleles	IC_50_	Antigenicity
ATDCSDGNY	211	A1 (3.5657)	HLA-A*01:01	102.93	0.7838
			HLA-C*12:03	6.55	
			HLA-C*05:01	31.89	
			HLA-C*14:02	205.8	
			HLA-B*15:02	82.46	
			HLA-A*30:02	83.84	
KLQPLTFLL	317	A2 (1.4454) A3 (0.7613) A24 (0.7629) B8 (0.8034)	HLA-A*02:01	18.79	1.14
			HLA-A*32:01	50.22	
			HLA-A*02:06	70.07	
			HLA-C*12:03	94.02	
			HLA-C*07:02	123.8	
LVRSESAAL	1086	B7 (1.5250) B8 (1.2136) B62 (0.9772)	HLA-C*12:03	84.41	0.41
			HLA-B*07:02	134.24	
			HLA-C*14:02	119.57	
			HLA-A*30:01	214.77	
			HLA-C*03:03	241.33	
MLKRRDSTY	696	A1 (0.8621) A3 (0.9329) B8 (1.7688) B62 (1.4024)	HLA-A*30:02	117.88	0.90
			HLA-B*08:01	55.78	
			HLA-B*15:01	63.24	
			HLA-C*12:03	6.67	
			HLA-C*14:02	153.33	
RRDSTYGPL	699	B27 (1.5815) B39 (1.1004)	HLA-C*07:02	41.60	1.42
			HLA-C*12:03	17.19	
			HLA-C*14:02	66.93	
			HLA-B*39:01	80.43	
			HLA-B*27:05	173.61	
LSIPTNFSF	780	B58 (2.112) B62 (1.39)	HLA-B*58:01	19.24	1.127
			HLA-B*15:01	44.15	
			HLA-A*23:01	194.72	
			HLA-C*03:03	49.73	
			HLA-C*12:03	56.52	
			HLA-B*57:01	236.49	
			HLA-B*35:01	156.16	
FSFGVTHEY	786	A1 (2.44) A3 (0.8719) A26 (1.4849) B62 (1.5080) B58 (1.71)	HLA-B*35:01	11.92	1.79
			HLA-B*58:01	94.25	
			HLA-A*30:02	131.05	
			HLA-A*01:01	230.96	
			HLA-C*07:01	21.94	
			HLA-C*12:03	9.27	
			HLA-A*29:02	25.22	
			HLA-B*46:01	47.2	
			HLA-A*68:01	93.86	
			HLA-B*15:01	159.59	

The epitopes listed in the table are non-allergic, non-toxic, and displayed 100% conservancy among target protein sequence (as predicted using the IDEB conservancy tool). Epitopes with half-maximal inhibitory concentration (IC_50_) values below 250 were considered to bind suitably with respective human leukocyte antigen (HLA) alleles. The VaxiJen v2.0 server was utilized at 0.4 thresholds to predict the antigenicity of the epitopes.

**Table 2:**
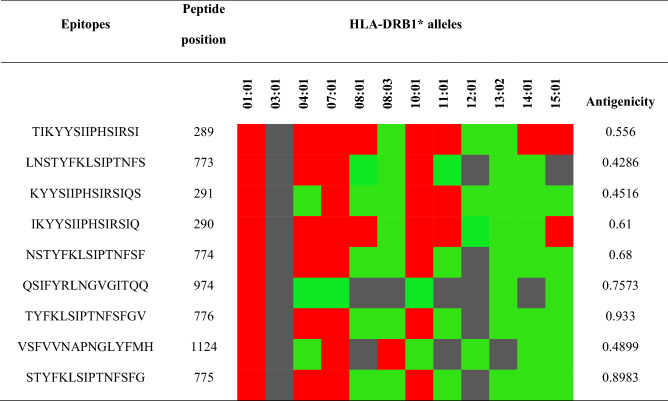
Selected helper T-lymphocyte epitopes, as predicted by the NetMHC II pan 3.2 server. The strong, intermediate, and non-binding affinities are represented by red, green, and black boxes, respectively. The epitope antigenicity was predicted by the VaxiJen v2.0 server at the 0.4-threshold level. The epitopes are promiscuous, non-allergic, non-toxic, and displayed 100% conservancy with the target sequence.

### Vaccine construction, structure modeling, refinement, and validation

**Figure 3:**
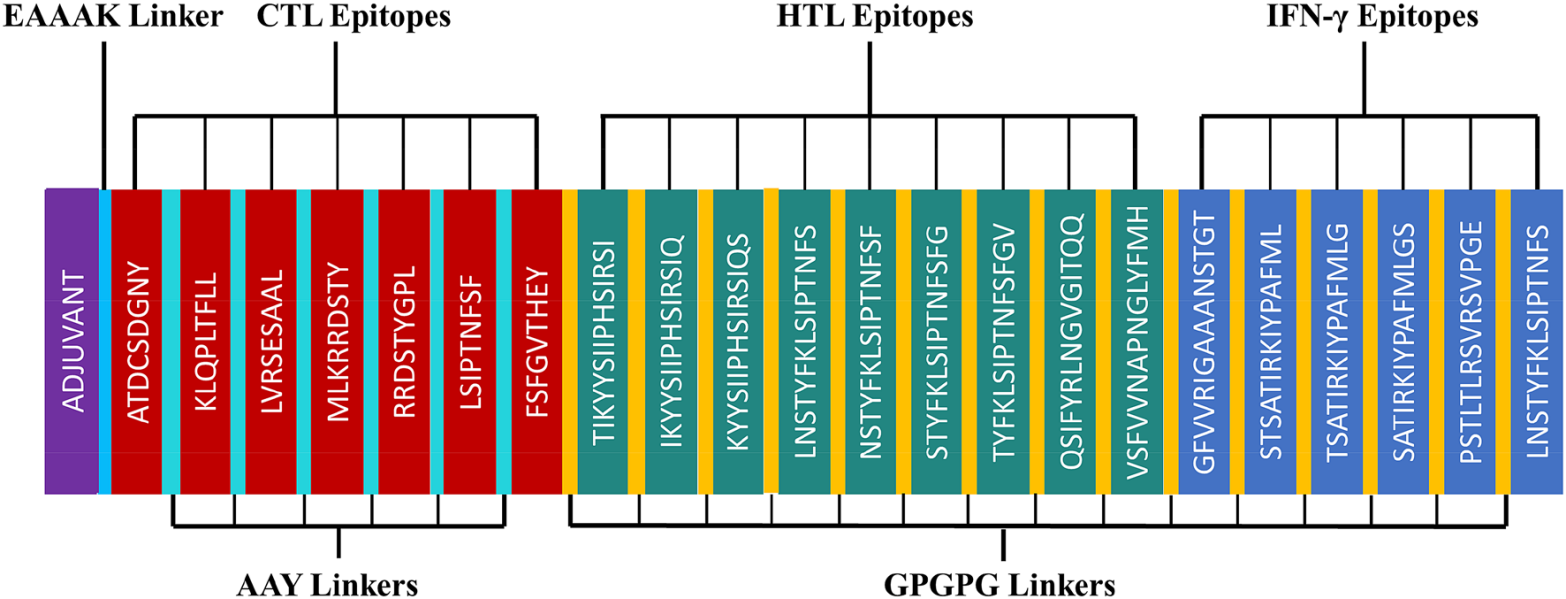
Schematic representation of the finalized vaccine construct that shows 489-amino acid long and containing CTL, HTL, and IFN-γ inducing epitopes, an adjuvant (violet) linked in the constructs via EAAAK linker (blue) at the end of N-terminal. CTL epitopes are linked with AAY linkers (green). HTL and IFN- γ epitopes are linked through GPGPG linkers (yellow).

**Figure 4:**
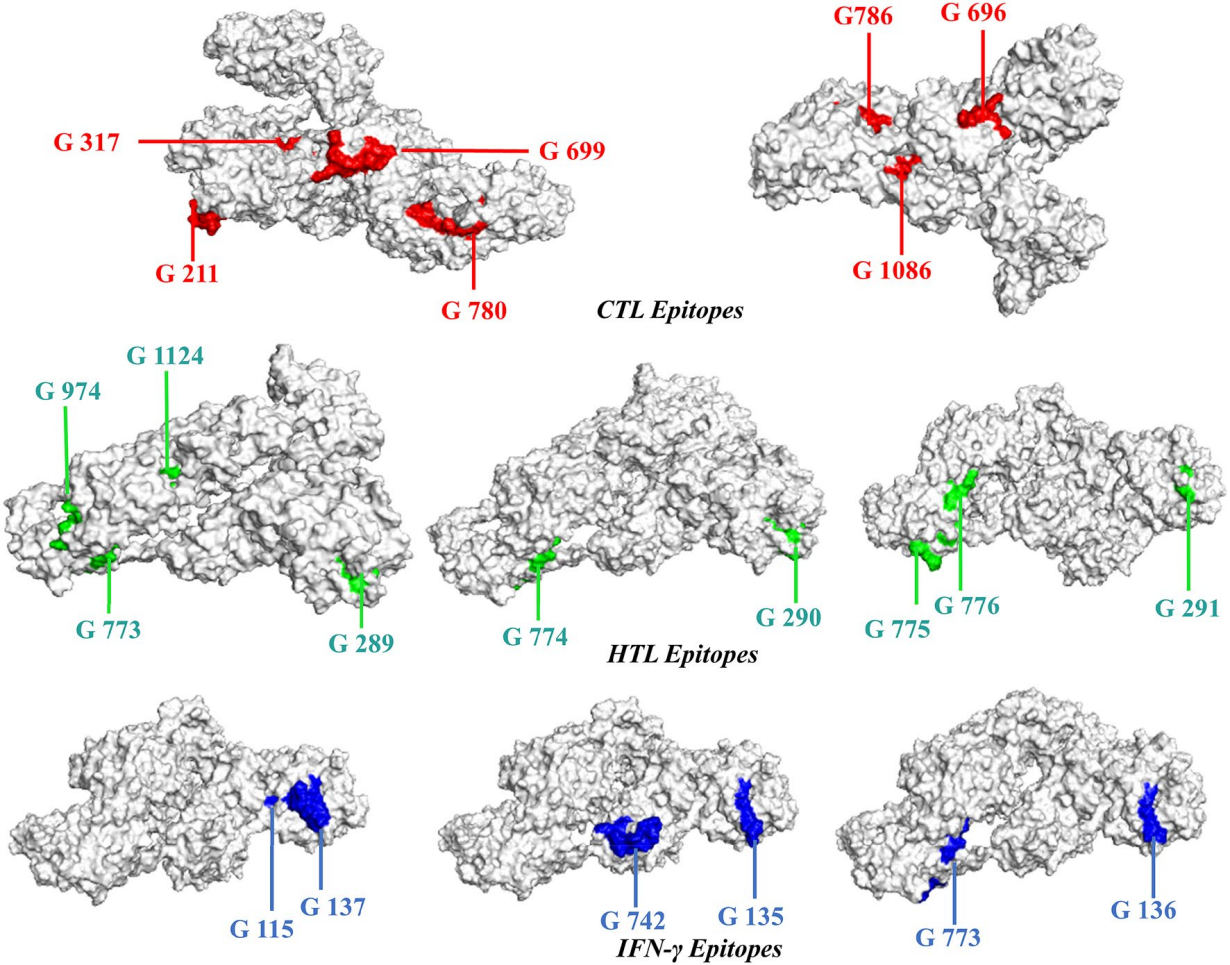
The surface view of the tertiary structure of spike glycoprotein with epitopes that are involved in the vaccine construct. The selected CTL, HTL, and IFN-γ epitope denoted in the structure by red, green, and blue colors, respectively.

**Figure 5:**
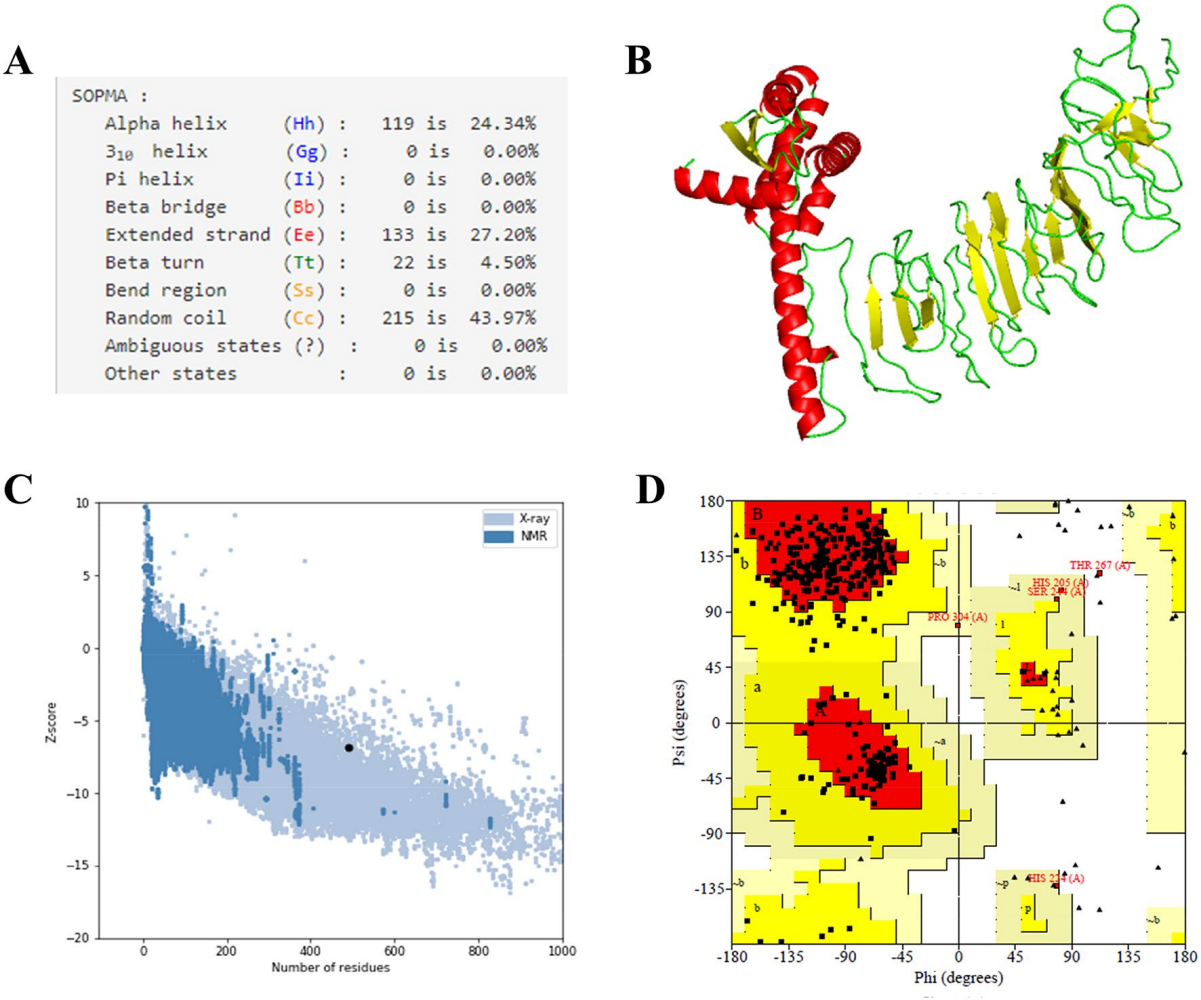
(**A**) The secondary structural properties of the vaccine. (**B**) 3-D model of the final vaccine. In this structure, red, yellow, and green color represents the helical, sheet, and loop regions, respectively. (**C**) 3-D structure validation with a Z-score of -6.85 followed by Pro-SA. (**D**) Analysis of Ramachandran plot utilizing PROCHECK server showed 87%, 12%, 0.8%, and 0.3% residues laying in favoured, additional allowed, allowed, and disallowed regions, respectively.

The criteria used for epitope selection during the construction of the linear vaccine included: (a) antigenic and non-allergic; (b) high-binding affinity with MHC alleles; (c) promiscuous; (d) containing overlapping CTL and HTL epitopes; (e) 100% conserved across S proteins; and (f) no overlapping capacity with any component of the human proteome. Epitopes that featured these properties were used to construct a linear vaccine sequence, comprising 7 CTL, 9 HTL, and 6 IFN-γ epitopes (Tables 1, 2, and Supplementary Tables 4–7). In addition, AYY and GPGPG linkers were added to the construction to prevent junctional epitope formation, facilitate the immune processing of the antigens and allow effective separation of each epitope within the human body^40^. To improve immunogenicity, cholera toxin subunit B (CTB) was added as an adjuvant attached to the N-terminal end (Fig. 3). The finalized vaccine sequence featured a molecular weight of 51.8 kDa and consisted of 489 amino acids (Supplementary Material SM1). The selected epitopes included in the vaccine were visualized on the 3D model of the S glycoprotein, which displayed the epitopes retaining their original positions (Fig. 4). The secondary structure prediction for the vaccine suggested that it would contain 24.34% alpha-helical regions, 27.20% extended strands, 4.50% beta turns, and 43.97% random coils (Fig. 5A). The 3D model of the finalized vaccine was prepared by the trRosetta web server which displayed five different models as per their Z-score ranging from 1.74 to 3.03 where the highest TM-score containing model (0.61 ± 0.05) was subjected to refinement. TM-score measures the similarity between two structures. A TM-score is higher than 0.5 is implying that a model of correct topology and less than 0.17 indicates random similarity^41^. Among all of the refined 3D structures, model 1 was selected which was predicted to be the best based on different parameters including MolProbity (2.727), GDT-HA (0.9059), and RMSD (0.519) (Fig. 5B and Supplementary Table 8). The poor rotamers and clash score of the finalized model were predicted to be 2.1 and 34.1, respectively. The global distance test-high accuracy (GDT-HA) is a global measure agreements between the experimental model and a predicted protein structure^42^. The Z-score of the finalized vaccine structure was − 6.85, as predicted by Pro-SA web (Fig. 5C), and the score was within the range for comparably-sized proteins, which indicated that the predicted model was reliable^43^. The results of the Ramachandran plot analysis showed that 87%, 12%, 0.8%, and 0.3% residues were in favoured, additionally allowed, allowed, and disallowed regions, respectively (Fig. 5D and Supplementary Materials SM2). The ERRAT value of the structure was predicted to be 56.017 (Supplementary Fig. S1). The ERRAT score higher than 50 indicating the good quality of a protein model^44^.

### Assessment of the antigenic, physicochemical, and allergenic profiles of the vaccine construct

The VaxiJen v2.0 server predicted a score of 0.5134, which confirms that the candidate vaccine is antigenic. Allergenicity was analyzed by AllerTOP v.2.0 and the AllergenFP v.1.0 server and was determined to be non-allergenic (Supplementary Fig. S2). The physicochemical properties of a vaccine must be determined to assess efficiency and safety^45^; therefore, several physical and chemical parameters of the vaccine construct were evaluated by ExPASy (Supplementary Materials SM3). The aliphatic index and theoretical pI values were found to be 71.10 and 9.74, respectively, which suggested that the candidate is thermostable. The estimated half-life was evaluated to be 30 h in mammalian reticulocytes, > 20 h in yeast, > 10 h in *E. coli*, The Grand average hydrophathicity (GRAVY) score is − 0.130 which specifies the candidate is hydrophilic in nature and has the ability to interact with the aqueous environment. The instability index was calculated as 26.58, which classified the protein as being stable^46^. In addition, our designed vaccine does not contain any signal peptides that would either specify or inhibit protein localization (Supplementary Fig. S3). The TMHMM server predicted that no production difficulties would be associated with expression (Supplementary Fig. S4).

### Post-translational modification analysis

A wide range of post-translational modifications was predicted in both targeted spike glycoprotein and the finalized vaccine construct. Analyzation of glycosylation site within the target protein indicated that there are two N-glycosylation sites were existing (position 6 and 244) and one O-GlcNAc site was found in 878 positions (Supplementary Figs. S5 and S6) which specifies the epitopes which are used in vaccine designing are not overlapping in those regions (PTM sites). Additionally, there was no lipid PTMs as N-terminal glycines myristoyl and GPI-modification was predicted in the finalized vaccine construct. Prediction of phosphorylation modification displayed 45 sites (Ser: 24, Thr: 16, Tyr: 5) in the construct. The YinOYang server predicted one O_GlcNAc attachment site to exist in the vaccine construct and there is no N-glycosylation and N-acetylation was found by NetNGlyc 1.0 server and NetAct 1.0 server, respectively (Supplementary Table 9).

### Prediction of the B-cell epitope

B-cells have significant roles in humoral immunity through the secretion of antibodies. A B-cell epitope can be recognized by the B-cell receptor, providing long-term immunity. The Ellipro server result included 10 linear and four conformational B-cell epitopes (Tables 3 and 4). PyMOL was used to visualize the mapped epitopes in the vaccine construct (Supplementary Figs. S7 and S8).

**Table 3:** Linear B-cell epitopes in the vaccine construct.

No	Start	End	Peptide	No. of residues	Score
1	427	489	MLGGPGPGSATIRKIYPAFMLGSGPGPGPSTLTLRSVRSVPGEGPGPGLNSTYFKLSIPTNFS	63	0.825
2	90	127	NNKTPHAIAAISMANEAAAKATDCSDGNYAAYKLQPLT	38	0.757
3	18	59	IHTLNDKIFSYTESLAGKREMAIITFKNGATFQVEVPGSQHI	42	0.753
4	145	170	YMLKRRDSTYAAYRRDSTYGPLAAYL	26	0.744
5	184	198	FGVTHEYGPGPGTIK	15	0.718
6	80	84	EAKVE	5	0.602
7	248	257	IQSGPGPGLN	10	0.597
8	385	398	ANSTTGPGPGSTSA	14	0.59
9	269	277	FSGPGPGNS	9	0.558
10	411	421	PGPGTSATIRK	11	0.544

**Table 4:** ElliPro server predicted a total of 259 residues, located in four discontinuous B-cell epitopes.

No	Residues	No. of residues	Score
1	A:M1, A:T2, A:P3, A:Q4, A:N5, A:I6, A:T7, A:D8, A:L9, A:C10, A:A11, A:H14, A:Q17, A:H19, A:T20, A:L21, A:N22, A:D23, A:K24, A:I25, A:F26, A:S27, A:Y28, A:T29, A:E30, A:S31, A:L32, A:A33, A:G34, A:K35, A:R36, A:E37, A:M38, A:A39, A:I40, A:I41, A:T42, A:F43, A:K44, A:N45, A:G46, A:A47, A:T48, A:F49, A:Q50, A:V51, A:E52, A:V53, A:P54, A:G55, A:S56, A:Q57, A:H58, A:I59, A:Q62, A:Y77, A:E80, A:A81, A:K82, A:V83, A:E84, A:C87, A:N90, A:N91, A:K92, A:T93, A:P94, A:I97, A:A98, A:A99, A:I100, A:S101, A:M102, A:A103, A:N104, A:E105, A:A106, A:A107, A:A108, A:K109, A:A110, A:T111, A:D112, A:C113, A:S114, A:D115, A:G116, A:N117, A:Y118, A:A119, A:A120, A:Y121, A:K122, A:L123, A:Q124, A:P125, A:L126, A:T127, A:T174	99	0.74
2	A:A385, A:N386, A:S387, A:T388, A:T389, A:G390, A:P391, A:G392, A:P393, A:G394, A:S395, A:T396, A:S397, A:A398, A:T399, A:I400, A:R401, A:K402, A:P411, A:G412, A:P413, A:G414, A:T415, A:S416, A:A417, A:T418, A:I419, A:R420, A:I422, A:Y423, A:P424, A:M427, A:L428, A:G429, A:G430, A:P431, A:G432, A:P433, A:G434, A:S435, A:A436, A:T437, A:I438, A:R439, A:K440, A:I441, A:Y442, A:P443, A:A444, A:F445, A:M446, A:L447, A:G448, A:S449, A:G450, A:P451, A:G452, A:P453, A:G454, A:P455, A:S456, A:T457, A:L458, A:T459, A:L460, A:R461, A:S462, A:V463, A:R464, A:S465, A:V466, A:P467, A:G468, A:E469, A:G470, A:P471, A:G472, A:P473, A:G474, A:L475, A:N476, A:S477, A:T478, A:Y479, A:F480, A:K481, A:L482, A:S483, A:I484, A:P485, A:T486, A:N487, A:F488, A:S489	94	0.734
3	A:Y145, A:M146, A:R149, A:R150, A:D151, A:S152, A:T153, A:Y154, A:A155, A:A156, A:Y157, A:R158, A:R159, A:D160, A:S161, A:T162, A:Y163, A:G164, A:P165, A:L166, A:A167, A:A168, A:Y169, A:L170, A:F184, A:G185, A:V186, A:T187, A:H188, A:E189, A:Y190, A:G191, A:P192, A:G193, A:P194, A:G195, A:T196, A:I197, A:K198, A:G213, A:P214, A:G215, A:I216, A:K217, A:P234, A:G235, A:K236	47	0.696
4	A:I248, A:Q249, A:S250, A:G251, A:P252, A:G253, A:P254, A:G255, A:L256, A:N257, A:F269, A:S270, A:G271, A:P272, A:G273, A:P274, A:G275, A:N276, A:S277	19	0.578

### Population coverage

Successful HLA allele calculations are necessary to develop a vaccine that will be effective for the entire global population. The IDEB Population Coverage tool indicated that the selected epitope included in our study would cover 100% of the worldwide population (Table 5 and Supplementary Table 10), with 100% coverage in East Asia, South Asia, Southwest Asia, North America, South America, and Europe. This result indicated that our designed vaccine could be used worldwide.

**Table 5:** Population coverage of the selected epitope included in the vaccine construct.

Population/area	Class combined		
	Coverage^a^	Average_hit^b^	pc90^c^
Central Africa	99.34%	26.31	14.8
East Africa	99.25%	25.78	14.64
East Asia	100.0%	39.72	28.42
Europe	100.0%	47.54	35.91
North Africa	99.8%	30.56	17.21
North America	100.0%	46.02	31.76
Northeast Asia	99.1%	24.76	14.4
Oceania	99.35%	24.71	14.5
South Africa	99.82%	28.55	15.78
South America	100.0%	31.57	21.27
South Asia	100.0%	40.36	28.65
Southeast Asia	100.0%	35.37	22.34
Southwest Asia	98.64%	23.09	14.02
West Africa	99.55%	27.53	15.21
West Indies	99.46%	24.44	14.66
World	100.0%	44.2	30.49
Average	95.83	30.82	19.71
Standard deviation	15.27	10.49	8.55

^a^Coverage of population on projected.

^b^Population recognized by HLA combinations/epitope hits on the average number.

^c^90% of the population recognized by HLA combinations/epitope hits on the minimum number.

### Analysis of molecular docking

**Figure 6:**
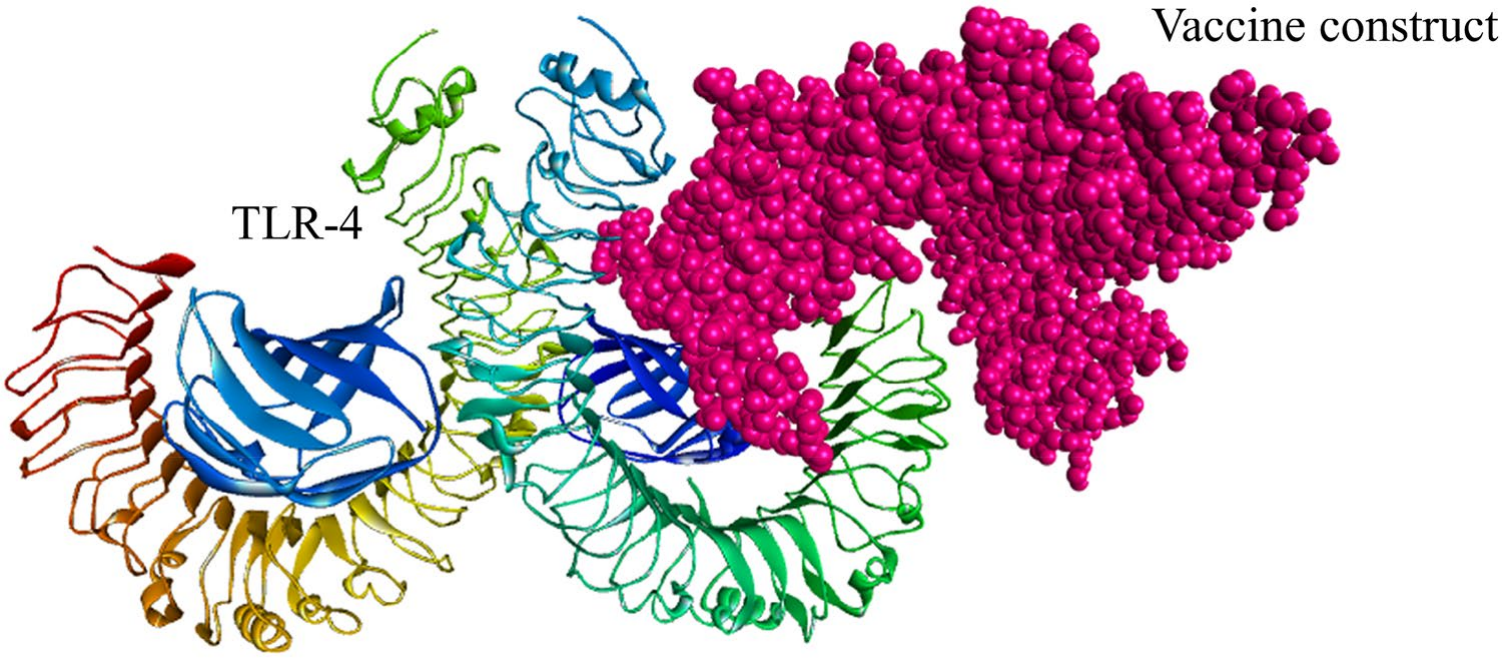
The docked complex of the designed multi-epitope vaccine protein and TLR-4 immune receptor. The vaccine construct has been shown in CPK molecular model (pink) in the figure and the global energy value of the finalized docked complex was − 21.16 kcal/mol.

**Figure 7:**
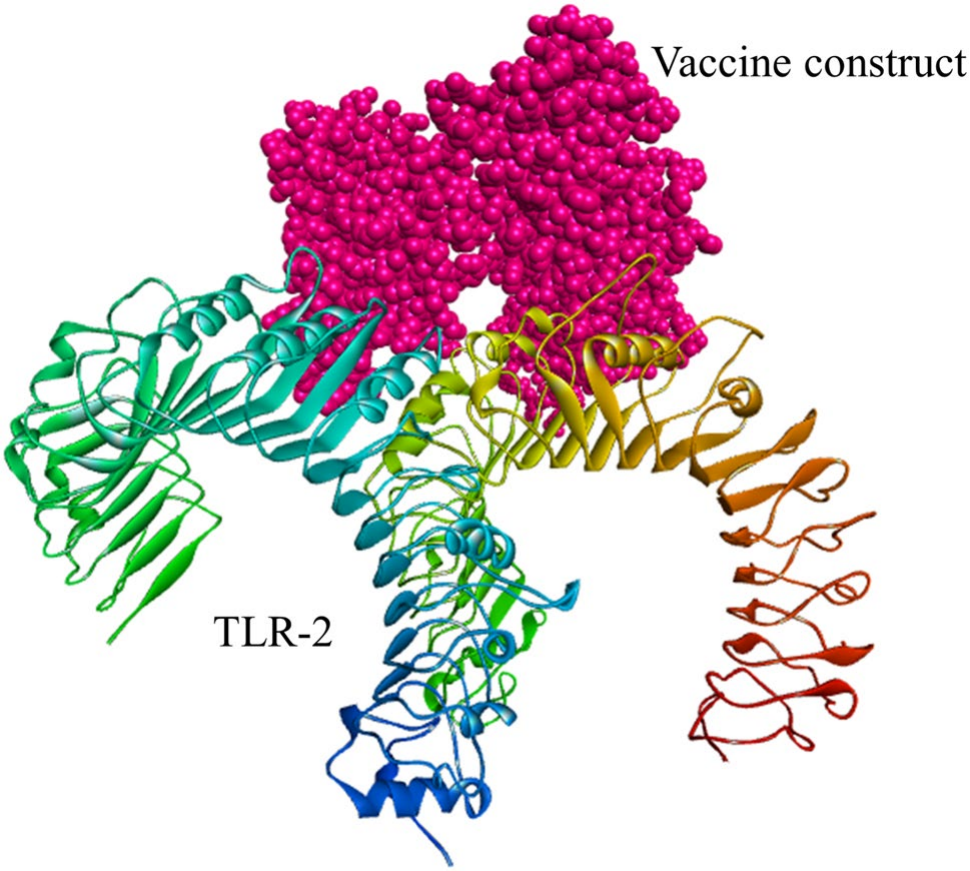
The docked complex of the designed multi-epitope vaccine protein and TLR-2 immune receptor. The vaccine construct has been shown in CPK molecular model (pink) in the figure. The global energy of the finalized docked complex was − 22.13 kcal/mol as predicted by the PatchDock server.

**Figure 8:**
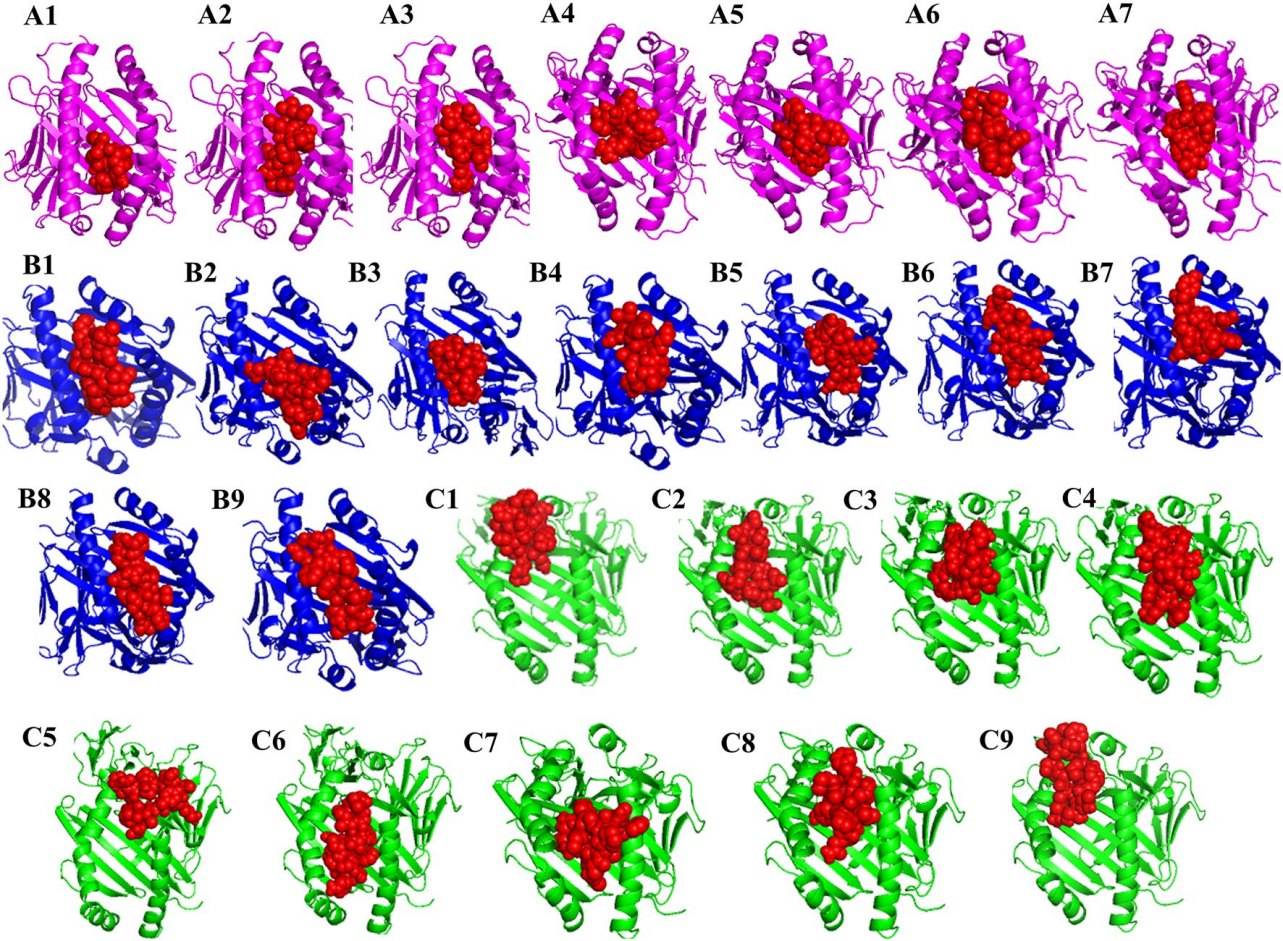
The interaction patterns of selected epitopes included in the vaccine constructs with HLA alleles. A1–A7 are the binding pattern of 7 CTL epitopes (red) with HLA-A*02:01 allele (magentas). The interaction pattern of 9 HTL epitopes (red) with the HLA-DRB1*01:01 (blue) allele has been shown in figure B1–B9. Figures C1–C9 represents the binding pattern of 9 HTL epitopes with HLA-DRB1*15:01 allele (green).

The interaction between immune cells and the vaccine is necessary for the development of stable immune response. Toll-like receptors (TLRs) serve as pathogen detectors and play crucial roles in innate immunity^47^. TLR2 and TLR4 can recognize viral structural glycoproteins, resulting in the production of inflammatory cytokines^48^. The molecular interactions pattern and binding affinities between the designed multi-epitope vaccine and the TLR-4 and TLR-2 immune receptors were analyzed via a protein–protein docking approach. Based on the electrostatic complementarity and geometry of the protein surface, the PatchDock server generates the interaction model of a receptor-ligand complex. The refinement of the best complexes was performed using the FireDock tool and found ten complexes for each TLR2-vaccine and TLR4-vaccine. Among the top-ten complexes generated when examining the interaction between the vaccine and TLR-4, solution 10 was characterized as having the best global energy (− 21.16), attractive van der Waals (VdW) energy (− 42.23), repulsive VdW energy (38.54), hydrogen bond (HB) energy (− 6.82), and atomic contact energy (ACE; 9.29) (Fig. 6 and Supplementary Table 11). Similarly, in the docking study between TLR-2 and the vaccine, the global energy (− 22.13), attractive VdW (− 34.45), repulsive VdW (26.29), HB (− 6.78), and ACE (23.41) values for solution 10 were better than those of the other models (Fig. 7 and Supplementary Table 12). In contrast, the selected CTL and HTL epitopes included in the finalized vaccine construct docked individually with three commonly occurring HLA alleles in the human population HLA-A*02:01, HLA-DRB1*01:01 and HLA-DRB1*15:01 (Fig. 8) and also compared the binding affinity with positive and negative control. We found that 7 CTL epitopes efficiently bind with HLA-A*02:01, the best interacting CTL model was KLQPLTFLL (lowest energy -795.6) as compared to the CTL positive (lowest energy -562.6) and negative (lowest energy 756.5) control (Supplementary Table 13). On the other hand, molecular interaction studies between HTL epitopes and HLA-DRB1 alleles have shown a strong binding affinity. The nine HTL epitopes were docked individually with HLA-DRB1*01:01 and HLA-DRB1*15:0, the best model was NSTYFKLSIPTNFSF, which shows the lowest energy (-987.0 with HLA-DRB1*01:01 and -1125.6 with HLA-DRB1*15:0) compared to the positive and negative control (Supplementary Table 14). Thus, these T-cell epitopes ensured their suitable binding affinity to be used in multi-epitope vaccine design against MERS-CoV.

### Analysis of molecular dynamics and simulation

**Figure 9:**
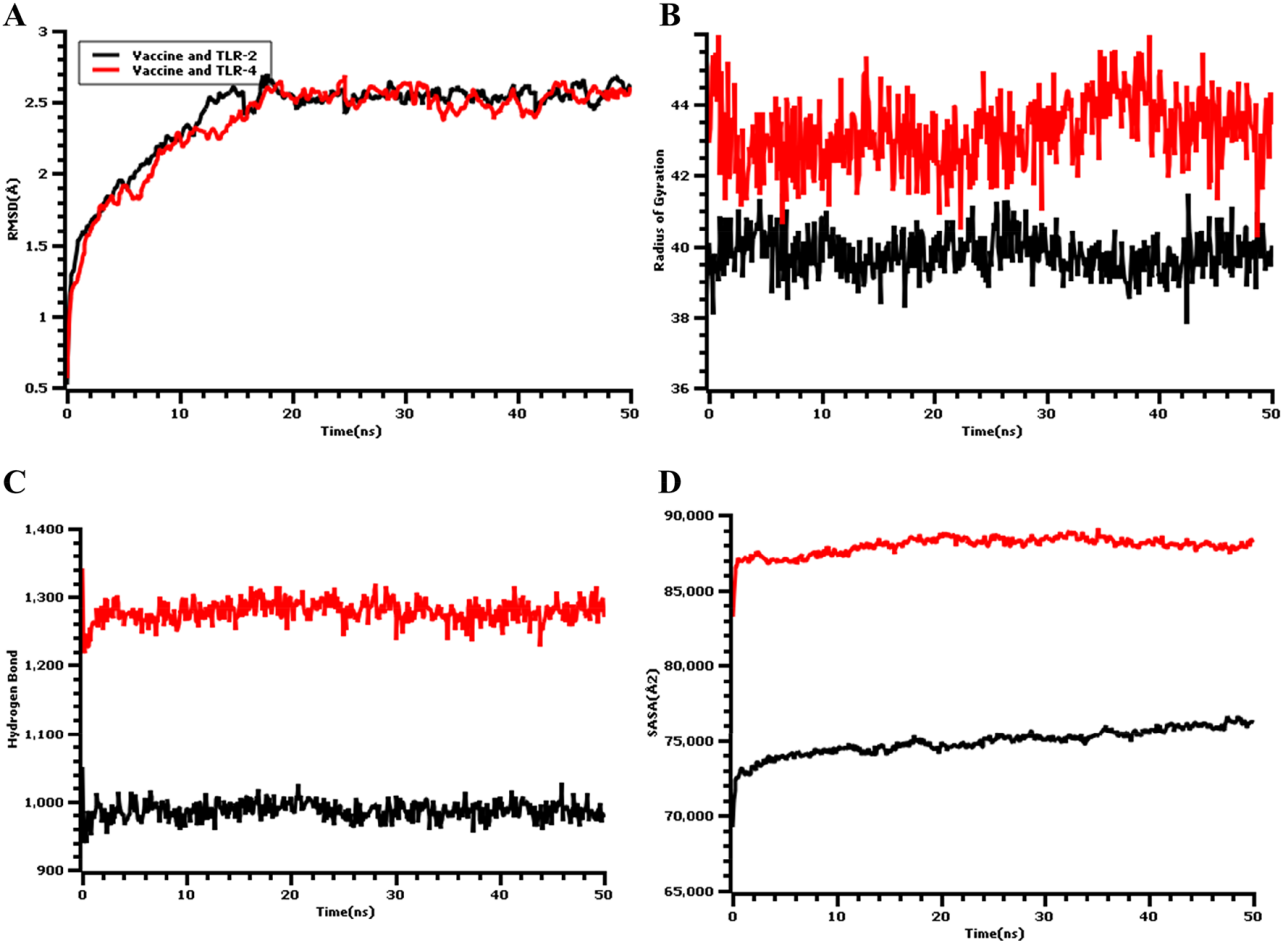
Molecular dynamic simulation result of the multi-epitope vaccine, and TLR-4 (red), TLR-2 (black) complexes at 50 ns. (**A**) The RMSD plot of the c-alpha atoms of the complexes. The mild fluctuations represent the stability of the complexes. (**B**) The Rg (Radius of Gyration) plot. (**C**) The Hydrogen bond (HB) plot of vaccine and TLRs docked complexes. (**D**) The SASA (Solvent-Accessible Surface Area) profile of the docked complexes.

**Figure 10:**
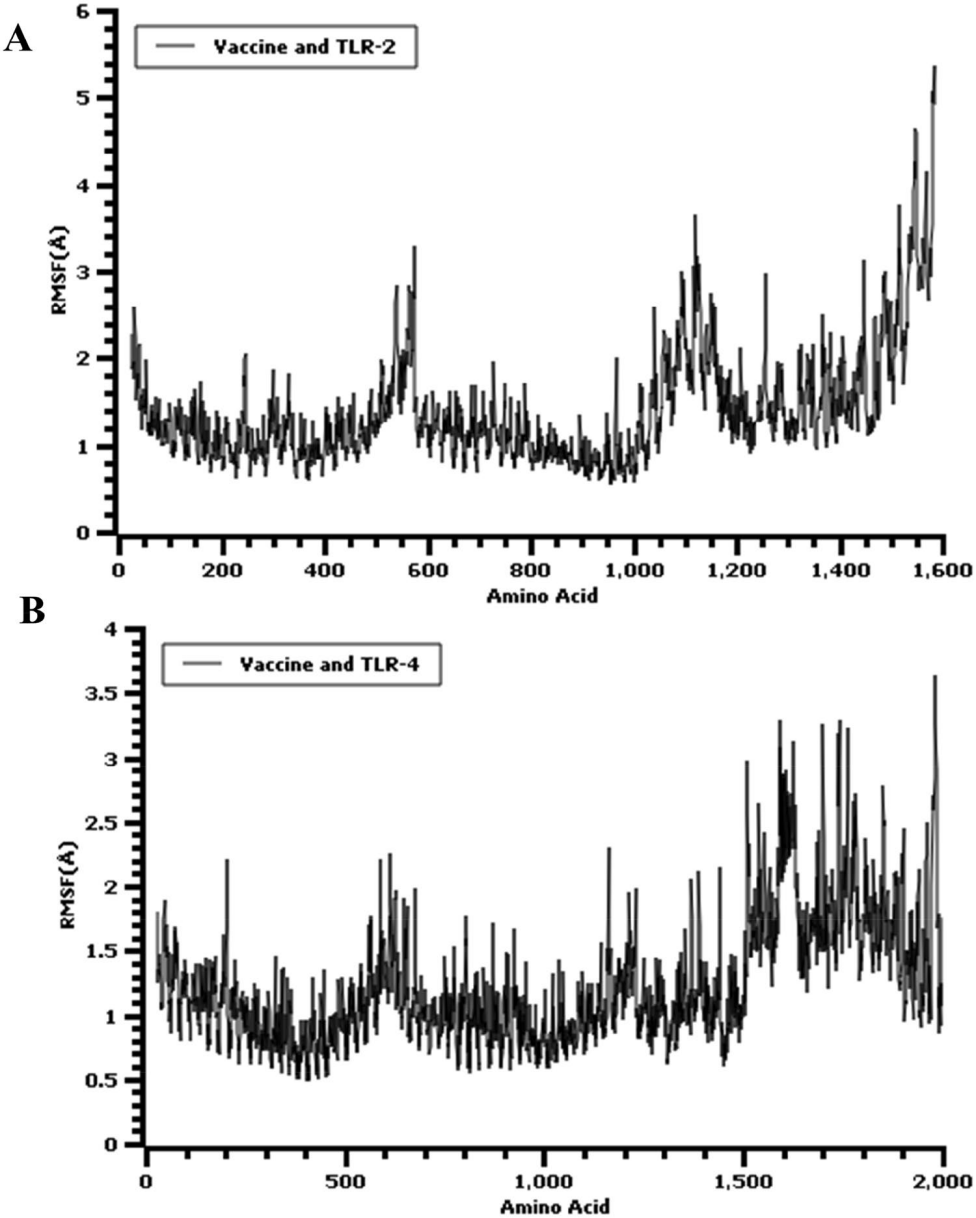
The RMSF plot of the multi-epitopic docked vaccine candidate and (**A**) TLR-2, (**B**) TLR-4 complexes at 50 ns.

The root means square deviation (RMSD) of the c-alpha atoms of the vaccine complex was calculated for two vaccine complexes. The average RMSD values for the vaccine and TLR-2 complex and for the vaccine and TLR-4 complex were 2.043 Å and 2.357 Å, respectively, which demonstrates the stable nature of both complexes. As shown in Fig. 9A, both the vaccine and TLR-2 and vaccine and TLR-4 complexes experience an initial increase in RMSD descriptors until the 15 ns time point, after which the upward trend ceases. A low degree of fluctuation was observed for both two complexes, which may be responsible for structural integrity. Therefore, the solvent-accessible surface area (SASA) values for both complexes were analyzed to understand changes in protein volume, based on the simulation trajectories. The average SASA value of the vaccine and TLR-2 complex was found to be 74,896.758Å^2^, which is similar to the entire SASA profile (Fig. 9D) because no deviations were found. In contrast, the vaccine and TLR-4 complex had an average SASA value of 87,958.090Å^2^. Both simulated complexes displayed an initial rise in the SASA values, which indicated the expansion of the protein volume during the initial phase. The radius of gyration (Rg) for the simulation trajectory provides information regarding the compressed nature of the protein, in which a higher Rg profile denotes less rigidity in the biological system. As shown in Fig. 9B, the Rg profile of the two vaccine complexes could be demonstrated, and an initial increase in the Rg profile for the vaccine and TLR-2 complex was observed. Therefore, the Rg descriptor trend for the vaccine and TLR-2 complex was similar from 0 to 50 ns, although a few fluctuations were observed. In contrast, the Rg value of the vaccine and TLR-4 complex was similar until 35 ns, followed by a larger deviation observed from 35–38 ns, which might be responsible for the loose packaging of the system. The vaccine molecules and their degrees of stability were further assessed by examining the total hydrogen bond number during the entire simulation period. The formation of hydrogen bonds and the number of substantial changes in the simulated complexes can be used to define the rigidity of the complexes. The vaccine and TLR-2 and the vaccine and TLR-4 complexes both featured stable numbers of hydrogen bonds, as shown in Fig. 9C, indicating relative complex stability. Moreover, the protein flexibility across the amino acid residues was evaluated by assessing the root mean square fluctuation (RMSF) profile. The RMSF profiles of the TLR-2 and TLR-4 complexes indicated that most of the amino acid residues from both complexes had RMSF profiles below 2.5 Å, and larger changes were only observed for a few residues. This result, as shown in Fig. 10, defines the vaccine complex stability and stiffness. Furthermore, secondary structure content from TLR-4-vaccine, TLR-2, and vaccine complexes was analyzed to understand their structural integrity (Supplementary Fig. S9). The secondary structure content; alpha helix, beta-sheet, coil and turn the region from both biological complexes were steady state in whole molecular dynamics simulation.

### In silico immune simulation

**Figure 11:**
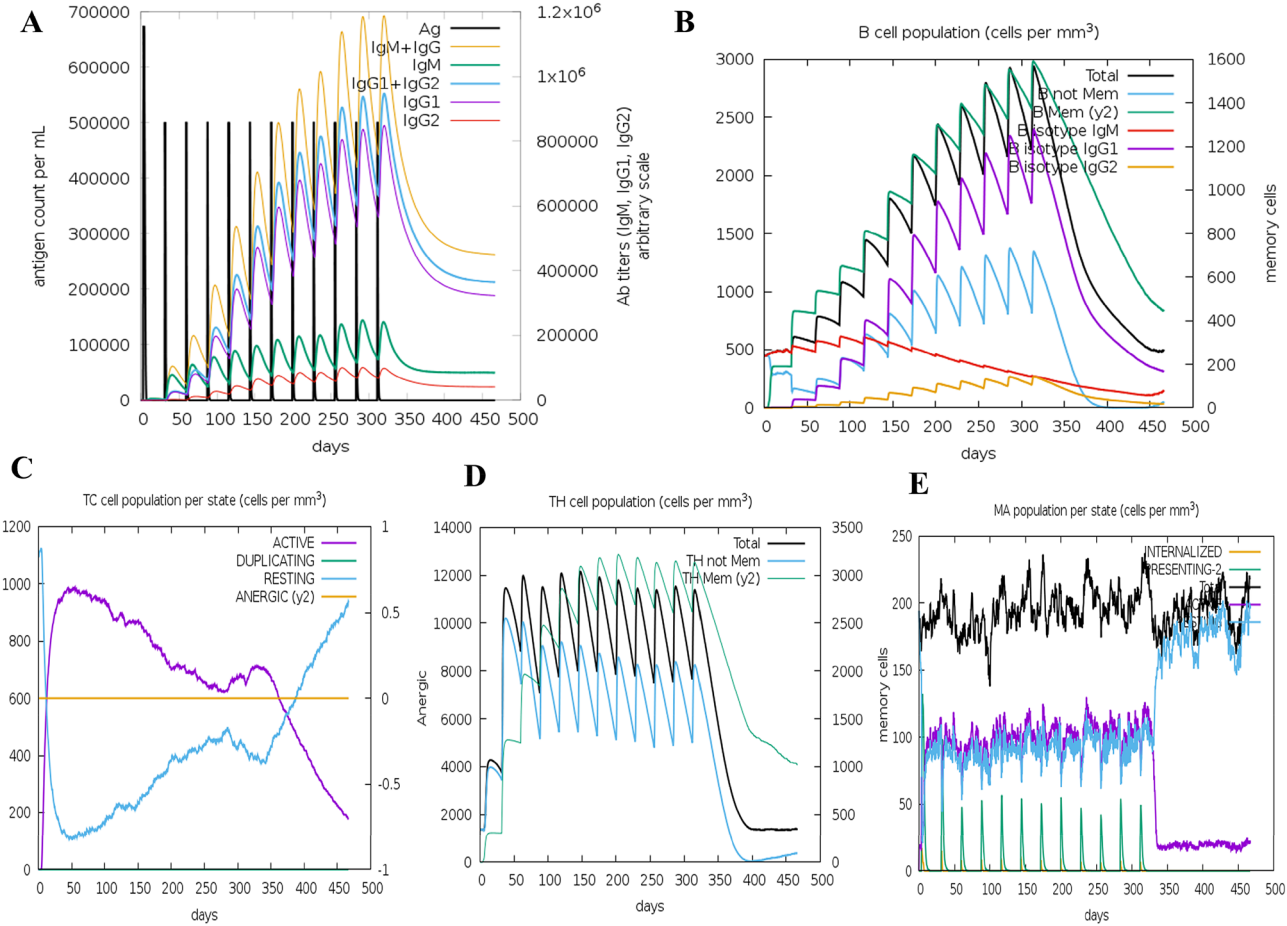
The in silico immune simulation profile of vaccine which injected 12 doses over a period of 12 months. (**A**) shows both antigen concentration and relative antibody responses. The efficacy of the vaccination showed by the presence of protective IgGs. (**B**) shows the corresponding count of antibody generating plasma cells while (**C−E**) show the activity of macrophages cytotoxic T, and helper T cells.

The immunogenic profile of the chimeric peptide vaccine is shown in Fig. 11 and Supplementary Fig. S10. The immune simulation results showed that the secondary and tertiary responses were considerably more immunogenic than the primary response. The antigenic concentration decreased, and the immunoglobulin activity became significantly increased during the secondary and tertiary responses (Fig. 11A). In addition, the formation of multiple B-cell isotypes was predicted (Fig. 11B). A similarly increased response was indicated by HTL and CTL populations during vaccination (Fig. 11C and D). Increased macrophage activity was detected in the form of natural killer (NK) and dendritic cells (Supplementary Fig. S10 and Fig. 11E). High levels of IFN-γ supported the activation of an adequate immune response, and IL-2 secretion was also elicited during the simulation (Supplementary Fig. S10E). This profile demonstrates the development of immune memory subsequently increased.

## Discussion

Although MERS is a highly infectious disease^49^ with a high mortality rate, no approved vaccines and or treatment drugs are currently available to prevent MERS-CoV infection. Vaccines are urgently necessary for the prevention and treatment of this disease. The application of an immunoinformatics strategy and the associated tools represents a rationally effective approach to the development of a peptide-based vaccine^26^. This approach is time-consuming, cost-effective, and can allow researchers to predict antigenic epitopes that may be potential candidates for use in an effective multi-epitope vaccine. In this investigation, our aim was to design a peptide-based poly-epitope vaccine against MERS-CoV. By applying a cutting-edge immunoinformatics strategy against the MERS-CoV proteome, the sequence of the most recognizable virulent factor, the S glycoprotein, was targeted. The antibody-mediated neutralization of the viral S protein is a primary goal in vaccine development because the spike glycoprotein is associated with viral attachment and entry to host cells. Here, we constructed a multi-epitope vaccine by using several computational tools. The vaccine was designed to provide immunity based on the use of several small antigenic peptide fragments; in contrast to vaccines that utilize the whole genome or large proteins, this approach does not produce any allergenic reactions in the host^50,51^. In addition, our designed vaccine has several advantages compared with conventional and single-epitope vaccines owing to the following distinctive characteristics: (a) the presence of multiple MHC epitopes, allowing the vaccine to be recognized by several T-cell receptors; (b) the use of overlapping CD4 + and CD8 + T-cell epitopes; (c) the presence of multiple epitopes from the targeted virulent protein; and (d) the inclusion of an immunostimulator (adjuvant) for producing long term immunity^52–55^. The design of a vaccine using a similar method has been demonstrated to result in the development of protective efficiency in vivo^56–58^, and some of these vaccines have entered the clinical trial phase^53,59–61^. The selection of antigenic epitopes is crucial for vaccine development^62^. The cytotoxic T-cell lymphocyte, helper T-cell lymphocyte, and interferon-gamma inducing epitopes of the S protein were identified based on the use of several immune filters. Epitopes were screened to identify antigenic but non-allergenic sequences that were capable of interacting with multiple HLA alleles were conserved 100% among the various S protein sequences and did not overlap with any components of the human proteome to reduce the possibility of autoimmunity. Through the application of various in silico analysis techniques, the designed poly-epitope (CTL, HTL, IFN-γ inducing)-containing vaccine was constructed with the addition of an N-terminal-linked Cholera toxin B (CTB) adjuvant (Fig. 3). In addition, GPGPG and AAY linkers were added to the sequences to prevent junctional epitope formation. Adjuvant and epitopes (CTL-HTL-IFN-γ) are ordered in such way have been shown to antigen-specific immune response stimulation reported in many studies^63,64^, the adjuvant is attached in the N-terminal of the construct because binding of it with TLR displays enhanced results in producing immune-response whereas TLRs associated to the activation of the humoral and cellular immunity^65^.

Cholera toxin B is potentially used as a viral adjuvant^66–68^. The use of specialized sequences including linkers has the ability to improve vaccine construct. Previously many studies demonstrated that GPGPG and AAY linkers^66,69^ were added between predicted HTL and CTL epitopes sequences respectively and produced junctional immunogenicity, consequently allowing the rational design construction of a potent poly-epitope vaccine^70^. Arai et al. reported that the EAAAK linker was incorporated between epitopes and adjuvant for improving bioactivity of fused protein and reaching a high level of expression and increasing the stability of the vaccine construct^71^. Similarly, Bazan and colleagues designed a T-cell-based multi-epitope subunit vaccine against the Ebola virus. They used the Immune Epitope Database (IDEB) to predict antigenic epitopes that were used to construct a vaccine candidate that was found to be immunogenic when expressed in mice^63^.

During vaccine development, allergenicity can be a major problem. In our final vaccine, allergenicity was not detected. Various physicochemical properties were determined using the ProtParam ExPASy tool, which indicated an instability index value of 26.58, which indicated that the vaccine would be stable. The theoretical PI value was calculated as 9.74. The aliphatic index of the vaccine was evaluated to be 71.10, which indicates that the protein would be thermostable. The GRAVY value was calculated to be − 0.130, which suggested that the vaccine can interact with water, has a polar nature, and a high degree of solubility. Foroutan and other researchers designed a vaccine and then validated their candidate through experimental evaluation, reporting that their vaccine was able to induce strong cellular and humoral immune responses in mice^64^. The aliphatic and instability index values for our designed vaccine were better than those reported for the vaccine candidate designed and tested by Foroutan et al. The Ramachandran plot analysis for the vaccine showed that 87%, 12.0%, 0.8%, and 0.3% residues were found in the favored, allowed, additionally allowed, and disallowed regions, respectively, with an ERRAT value and a Z-score of 56.017 and − 6.85, respectively, which indicates that the protein falls in the plot which consists of the Z-scores of the already determined structures solved by NMR and X-ray crystallographic experiments^43^. TLR-4 is expressed in monocytes, granulocytes, and immature dendritic and macrophage cells^72^. The direct interaction between CTB and TLR-4 facilitates the activation of TLR-4 by CTB^73^. An enzyme-linked immunosorbent assay (ELISA) indicated that CTB is capable of inducing the activation of NF-κB in TLR-4 receptor cells through direct binding^73^. The viral E glycoprotein can also be recognized by TLR-2^74^. Molecular docking studies were used to analyze the molecular interactions and binding affinity patterns between the vaccine and both TLR-2 and TLR-4. The global energies of the best-docked complexes between the vaccine and TLR-4 and TLR-2 were − 21.16 kj/mol and − 22.13 kJ/mol, respectively, which denoted favorable binding affinities. The molecular dynamics simulation study of the vaccine candidate and TLR-4 and TLR-2 complexes were conducted to confirm their stable nature at atomistic conditions. The simulation data by combining RMSD, RMSF, SASA, Rg descriptors from trajectories correlates with the structural rigidity of the vaccine complexes. The RMSD and RMSF profile of the vaccine candidates were below 2.5 Å for most of the simulation time. These results define the vaccine complexes' integrity and less mobility at the simulation conditions. The immune simulation study indicated that our designed vaccine candidate could likely generate an appropriate immune response during secondary exposure after the final injection (Fig. 11). Many researchers have recently applied similar immunoinformatics approaches to the design of multi-epitopic vaccine candidates against Kaposi’s sarcoma^26^*, Klebsiella pneumoniae*^75^, *Pseudomonas aeruginosa*^76^, dengue^69^, Nipah virus^77^, SARS-CoV-2^78^, Hendra virus^79^, and malaria^80^. A similar strategy has also been used to develop a vaccine against cancerous antigens^81^. Therefore, the construction of a vaccine using epitopes appears to be capable of inducing the activation of immune cells in the host, which may further trigger other immune cells via a complex signaling pathway.

## Materials and methods

### Retrieval of protein sequence and the analysis of the phylogenetic tree

The NCBI database was utilized to retrieve the S glycoprotein amino acid sequences for MERS-CoV (Accession no: ANI69889.1), which were stored in a FASTA format. The sequences were aligned using the MUSCLE tool^82^, and a phylogenetic tree was constructed by using MEGA-X^83^. The Jones-Taylor-Thornton (JTT) model was used to an estimated algorithm for a matrix of pairwise distances to select superior log-likelihood values.

### Antigenic and physiochemical evaluation of the target protein

The prediction of antigenicity is an important step when attempting to isolate the most antigenic protein sequences. The stored protein sequence was submitted to the VaxiJen v2.0 Server^84^ in plain sequence format to determine antigenicity, using default parameters. The physicochemical properties of the protein were analyzed by the ExPASy ProtParam tool^46^.

### T-cell epitope prediction and assessment

#### CTL epitope prediction

Cytotoxic (CD8^+^) T-cell epitopes were predicted by submitting FASTA sequences of the target protein to the NetCTL-1.2^85^ server where thresholds were set for epitope identification, TAP transport efficiency, and proteasomal C-terminal cleavage were 0.75, 0.05, and 0.15, respectively. NetCTL 1.2 can predict epitope from the query sequence on the basis of the training dataset. The CTL epitopes were predicted by recognizing the commonly occurring HLA 12 Class I supertypes and further IEDB SMM method was used to evaluate the binding affinities of the epitopes with MHC class I alleles^86^. Each of the output epitope from the IDEB server is assigned as IC50 values which characterize the binding affinity of peptide molecules in the MHC allele. The IC50 values < 50 nM specifies high-binding affinity, lesser than 500 nM categorize intermediate affinity, whereas < 5000 nM indicate low affinity^66^. Epitopes with binding affinities below 250 nM (IC_50_) were chosen for further analyses.

### HTL epitope prediction

The Net MHCII pan 3.2^87^ server was utilized to predict 15-mer-long epitopes capable of recognizing human leukocyte antigen class II DRB1 alleles:01:01, 03:01, 04:01, 07:01, 08:01, 08:03, 10:01, 11:02, 12:01, 13:02, 14:01, and 15:01. The server uses artificial neuron networks to predicts the peptide that binding to HLA-DQ, HLA-DR, and HLA-DP alleles, and the prediction of epitopes was performed based on receptor affinity, which is assigned a percentile rank for each predicted output. The predicted epitopes were divided into strong, weak, and non-binder, based on percentile scores of less than 2%, 2%–10%, and greater than 10%, respectively. The VaxiJen v.2.0 server^84^ was used for assessing the antigenicity of each epitope at 0.4 thresholds. AllerTOP v.2.0^88^, AllergenFP v.1.0^89^, AllerCatPro v.1.7, and ToxinPred server were applied to screen out epitopes based on non-allergenicity and non-toxicity. The visualization of the epitope in the S glycoprotein was performed using PyMOL.

### Prediction of B-cell epitopes

Linear and conformational B-cell epitopes were predicted by the ElliPro server^90^ of IDEB. This method is driven by three algorithms that perform the calculation of the protein shape as an ellipsoid, the residues protrusion index (PI) calculation, and neighboring residues clustering on the basis of PI values. For the output of each B-cell epitope, ElliPro gives a score described as PI values for each residues. The residue Protrusion Index (PI) was also calculated. ElliPro indicated that 90% of protein residues were associated with PI values of 0.9 and 10% of residues contain without ElliPsoid. Ellipsoid and ElliPro are considered the most significant prediction tools for all proteins^90^.

### Prediction of interferon-gamma inducing epitopes

In both cell-mediated and adaptive immune systems, interferon-gamma (IFN-γ) cytokine plays a significant role to stimulate natural killer cells and macrophages for exerting immunity against viral and bacterial infections. For the prediction of IFN-γ in the target protein, we used the IFN epitope server (http://crdd.osdd.net/raghava/ifnepitope/)^91^, which is based on an IFN-γ dataset. The main aim of this server is to design and predict IFN-γ inducing capacity containing peptide sequences from the query protein and it works based on a training dataset of 10,433 experimentally validated helper T-cell epitopes from the IDEB database^91^. From the input protein, the server generates overlapping IFN-γ inducing epitopes as displayed by the numerical score. The support vector machine (SVM) and a motif hybrid method were used to perform the prediction.

### Population coverage and epitope conservancy calculation

The expression and distribution of HLA alleles could vary throughout the world according to the difference in regions. Thus, it is necessary to assess the HLA allele distribution around the world population. population coverage of the selected epitope was analyzed by the IEDB population coverage tool^92^ and the conservancy of each epitope was evaluated by the IEDB analysis resource.

### Molecular interaction pattern analysis of selected epitopes with HLA alleles

The three-dimensional structure of selected CTL and HTL epitopes was generated by an online server named PEPFOLD 3.5^93^ and the 3D crystal structure of the three commonly occurring HLA alleles in the human population HLA-A*02:01, HLA-DRB1*01:01, and HLA-DRB1*15:01 was downloaded from the RCSB PDB database ID- 1QEW, 2G9H, and 1BX2, respectively. The HLA class I and class II alleles used in the docking study are expected to cover more than 95% of the worldwide population^94^. The PDB structure was prepared before running P-P docking by removing water and ligand and energy minimization of the structure was carried out. In order to analyze the interaction pattern of screened out best epitopes with HLA alleles, ClusPro 2.0^95^ P-P docking server was employed. This web-based docking tool accomplishes rigid docking by sampling billions of conformations, energy minimization, and pairwise RMSD of the complexes and estimate binding energy score of the Protein–Protein docked complex on the basis of shaped complementarity, Deocys as references states, and desolvation contribution. Molecular docking was carried out under the hydrophobic environment and the best cluster (complex) of epitopes and alleles was selected based on the lowest docking energy score. Finally, PyMol was used to visualize the interaction between epitopes and alleles.

### Vaccine construction, structure modeling, and validation

The selected CTL, HTL, and IFN-γ epitopes were linked by using AYY and GPGPG linkers, and an adjuvant CTB was added to the N-terminal of the sequences via an EAAAK linker. Secondary structural features of the vaccine construct including extended strands, alpha-helical regions, random coils, and beta turns were predicted by the SOPMA server. The trRosetta (transform-restrained Restta) online tool^96^ (https://yanglab.nankai.edu.cn/trRosetta/) was applied to generate the three dimensional model of the linear vaccine constructs, and the GalaxyRefine web server^97^ was used to refine the model. The trRoseta server has been considered to be the accurate and fast algorithms for de novo protein structure prediction and the principle of this algorithm is to attempt to mimic the interplay of global and local interaction in defining protein model. On the basis of direct energy minimizations with a retrained Rosetta this computational tool generates three dimensional structure from the input protein sequence. The restrained comprises orientation distributions and inter-residues distance^98^. Galaxy Refine web server primarily reconstructs side chains and accomplishes repacking of the side chain and then utilizes molecular dynamic simulation to attain overall structure relaxation. This algorithm improves the quality of local structure according to CASP10 calculation techniques^99^. Validation of the vaccine structure was performed based on the ERRAT and Z-score^43^. ProSA-web determines and assigns Z-score for input protein structure which is shown on the plot in the context of all known protein models whereas structures have been evaluated by X-ray crystallography and NMR, this tool also displayed any problematic part of the structure as highlighted in three-dimensional molecule viewer. When the score was outside the range for comparably-sized proteins it specifies structure error^43^. The ERRAT web server evaluates non-bonded atom–atom interaction compared to reliable high-resolution crystallography structures. Finally, the overall structural quality was validated by a Ramachandran plot analysis, followed by the PROCHECK server^100^.

### Assessment of the physicochemical and antigenic properties of the vaccine

Antigenicity was measured by the VaxiJen v2.0 web tool^84^, where allergenicity was checked by the AllerTOP v.2.0^88^ and AllergenFP v.1.0^89^ tools. VaxiJen v2.0 is a freely available server that can predict the antigenicity of a sequence on the basis of auto- and cross-covariance (ACC) transformation of protein sequences into uniform vectors of principal amino acid properties. AllerTOP v2.0 classifies the allergens of a protein using amino acid E-descriptors, k nearest neighbor’s machine learning methods, and the auto- and cross-covariance (ACC) transformation. Alternatively, AllergenFP is a descriptor-based and alignment-free strategy to recognize allergens and non-allergens. It is essential to determine physical and chemical parameters associated with the vaccine construct. Therefore,various physicochemical properties of the vaccine like instability index, aliphatic index, molecular weight, GRAVY values, isoelectric point, and half-life were evaluated by using the ExPASy ProtParam tool^46^. SignalP 4.1^101^ and TMHMM v2.0^102^ servers were used to check any transmembrane helices in the vaccine and the existence of any signal peptides.

### Analysis of post-translational modification

In order to analyze post-translational modification in both the target spike glycoprotein and the designed vaccine construct including glycosylation, phosphorylation, and acetylation, we have used YinOYang 1.2, NetNGlyc 1.0, NetPhos 3.0, and NetAct 1.0 servers which are available at http://www.cbs.dtu.dk/services. The NetPhos 3.2 web tool can predict phosphorylation sites at threonine, tyrosine, and serine for amino acid sequences^103^. The NetNGlyc and YinOYang server use an artificial neural network to predict N-terminal glycosylation and O_GlcNAc attachment sites in mammalian protein^104^.

### Molecular docking and refinement

To calculate the binding affinity and interaction patterns between the designed multi-epitope vaccine and Toll-like receptor 2 (PDB: 2Z7X) and Toll-like receptor 4 (PDB: 3FXI), the structures were retrieved from the RCSB PDB database in pdb format. The PatchDock server^105^ was used to perform molecular docking, and the refinement of the best complex was performed by the FireDock server^106^. PatchDock server calculates surface fix coordinating scores, separating scores, and portrayal of atomic shape for protein–protein molecular docking. This algorithm splits the TLRs receptor and the vaccine molecules into small patches in agreement with the surface. These small patches resemble distinctive shapes, which can separate puzzle pieces visually. The lowest docking energy score was used to select the top-ranked vaccine-TLRs complex structure. In addition, molecular interactions were visualized through PyMol and Discovery Studio 2020.

### Molecular dynamic simulation

The YASARA software package^107^ was implemented to perform molecular dynamics simulations of the vaccine/TLR complexes. The AMBER14 force field^108^ was used for the system, and the vaccine complex was initially cleaned and optimized for hydrogen bond formation. The simulation system was established with the aid of a cubic simulation cell. For the initial energy minimization process, a simulated annealing method was applied with steep gradient approaches. The system was neutralized by the addition of water molecules and 0.9% NaCl salt. The physiological conditions of the system were set to pH 7.4 and a temperature of 310 K^109^. The system temperature was maintained with a Berendsen thermostat. The Part6icle Mesh Ewald (PME) method was applied for the calculation of long-range electrostatic interactions and short-range Columb and vdW interactions. A cut-off radius was established at 8 Å^110^. The simulation time step was set to 1.25 fs, and simulation trajectories were saved after every 100-ps interval^107^. Finally, the simulation was performed for 50 ns, and the RMSF, RMSD, Rg, and SASA values and the numbers of hydrogen bonds formed during the trajectories were analyzed to reveal the stabilities of the vaccine complexes^111–115^.

### In silico immune simulation

The C-IMMSIM webserver was utilized to perform immune stimulations, assess immunogenicity, and determine the immune response profile for our vaccine^116^. This server is an agent-based model that uses position-specific scoring matrices to predict peptides derived from machine learning techniques for predicting immune interactions. It simulates three compartments which are three anatomical regions in mammals: (1) the bone marrow (the simulation region of hematopoietic stem cell for producing new myeloid and lymphoid cells); (2) the thymus (where native T-cells are selected to avoid autoimmunity); and (3) lymph node^116^. The minimum recommended time between dose 1 and dose 2 for most of the vaccines currently in use, is 4 weeks^117^. The entire simulation ran for 1,400-time steps which are about 15 months (a time step is about 8 h). Two peptide injections were given four weeks apart at time step 10, 94, 178, 262, 346, 430, 514, 598, 682, 766, 850, 934.

## Conclusions

Multi-epitope vaccines have already gained importance and demonstrated protective efficiency, capable of generating immunity in vivo, with some entering clinical trials. The present study was based on an immunoinformatics-driven method that was used to identify potential antigenic epitopes for use in a vaccine candidate against MERS-CoV. Three antigen categories, including CTL, HTL, and IFN-γ epitopes, of the S glycoprotein, were used to construct a multi-epitope vaccine. The physicochemical and antigenic profiles of the vaccine were studied computationally. The stability profile and molecular interactions between the designed vaccine and immune receptors were assessed through molecular dynamics simulations and molecular docking studies. In silico immune simulation indicated the vaccine’s ability to trigger an immune response. A series of immunoinformatics strategies were applied sequentially to design and evaluation of a vaccine that may produce a protective immunity against viral infection, however, the experimental evaluation is required to assess the exact efficiency. The experimental assay may include the synthesis of the vaccine followed by the in vitro and in vivo assay. Additionally, we propose more studies that include synthesis and biological activities of the designed multi-epitope vaccine.

## Supplementary Information

Supplementary Information.

## Data Availability

All data generated or analysed during this study are included in this published article (and its Supplementary Information files).
