# Supplementary material for: Designing a multi-epitope vaccine candidate to combat MERS-CoV by employing an immunoinformatics approach
Source: Sci Rep. 2021 Jul 29;11:15431. doi: 10.1038/s41598-021-92176-1 (PMC8322212; doi:10.1038/s41598-021-92176-1)
Supplement: Supplementary file 1 — Supplementary Information. [file 41598_2021_92176_MOESM1_ESM.docx]

**Designing a multi-epitope vaccine candidate to combat MERS-CoV by employing an immunoinformatics approach**

**Shafi Mahmud ^1,^**^†^**, Md. Oliullah Rafi ^2,^**^†^**, Gobindo Kumar Paul ^1^, Maria Meha Promi ^3^, Mst. Sharmin Sultana Shimu ^3^, Suvro Biswas ^3^, Talha Bin Emran ^4^, Kuldeep Dhama ^5^, Salem A. Alyami ^7^, Mohammad Ali Moni ^6,*^ and Md. Abu Saleh ^1*^**

*^1^Microbiology Laboratory, Department of Genetic Engineering and Biotechnology, University of Rajshahi, Rajshahi-6505, Bangladesh*

*^2^Department of Genetic Engineering and Biotechnology, Jashore University of Science and Technology, Jashore-7408, Bangladesh*

*^3^Department of Genetic Engineering and Biotechnology, University of Rajshahi, Rajshahi-6505, Bangladesh*

*^4^Department of Pharmacy, BGC Trust University Bangladesh, Chittagong-4381, Bangladesh*

*^5^Division of Pathology, ICAR-Indian Veterinary Research Institute, Izatnagar, Bareilly-243122, Uttar Pradesh, India*

*^6^WHO Collaborating Centre on eHealth, UNSW Digital Health, School of Public Health and Community Medicine, Faculty of Medicine, UNSW Sydney, NSW 2052, Australia*

*^7^Department of Mathematics and Statistics, Imam Mohammad Ibn Saud Islamic University, Riyadh, 11432, Saudi Arabia*

^†^ These authors contributed equally to this work

***Corresponding Author:** saleh@ru.ac.bd and m.moni@unsw.edu.au

**SUPPLEMENTARY MATERIALS**

**Supplementary Materials SM 1:** Primary sequence of the final vaccine construct.

**MTPQNITDLCAEYHNTQIHTLNDKIFSYTESLAGKREMAIITFKNGATFQVEVPGSQHIDSQKKAIERMKDTLRIAYLTEAKVEKLCVWNNKTPHAIAAISMAN**EAAAKATDCSDGNYAAYKLQPLTFLLAAYLVRSESAALAAYMLKRRDSTYAAYRRDSTYGPLAAYLSIPTNFSFAAYFSFGVTHEYGPGPGTIKYYSIIPHSIRSIGPGPGIKYYSIIPHSIRSIQGPGPGKYYSIIPHSIRSIQSGPGPGLNSTYFKLSIPTNFSGPGPGNSTYFKLSIPTNFSFGPGPGSTYFKLSIPTNFSFGGPGPGTYFKLSIPTNFSFGVGPGPGQSIFYRLNGVGITQQGPGPGVSFVVNAPNGLYFMHGPGPGGFVVRIGAAANSTTGPGPGSTSATIRKIYPAFMLGPGPGTSATIRKIYPAFMLGGPGPGSATIRKIYPAFMLGSGPGPGPSTLTLRSVRSVPGEGPGPGLNSTYFKLSIPTNFS

**Bold Text** = Adjuvant

EAAAK, AAY, GPGPG = Linkers

9 mer epitopes = CTL epitopes

15 mer epitopes = HTL and IFN-γ epitopes

**Supplementary Materials SM 2:** Ramachandran plot statistics of the vaccine structure.

**Plot statistics**

Residues in most favoured regions [A, B, L] 327 87.0%

Residues in additional allowed regions [a,b,l,p] 45 12.0%

Residues in generously allowed regions [~a,~b,~l,~p] 3 0.8%

Residues in disallowed regions 1 0.3%

---- ------

Number of non-glycine and non-proline residues 376 100.0%

Number of end-residues (excl. Gly and Pro) 2

Number of glycine residues (shown as triangles) 61

Number of proline residues 50

----

Total number of residues 489

Based on an analysis of 118 structures of resolution of at least 2.0 Angstroms

and R-factor no greater than 20%, a good quality model would be expected

to have over 90% in the most favoured regions.

**Supplementary Material SM 3:** Physico-chemical properties of the final multi-epitope vaccine construct.

**Number of amino acids**: 489

**Molecular weight**: 51898.22

**Theoretical pI**: 9.74

**Amino acid composition**

Ala (A) 39 8.0%

Arg (R) 18 3.7%

Asn (N) 21 4.3%

Asp (D) 8 1.6%

Cys (C) 3 0.6%

Gln (Q) 11 2.2%

Glu (E) 11 2.2%

Gly (G) 61 12.5%

His (H) 9 1.8%

Ile (I) 39 8.0%

Leu (L) 29 5.9%

Lys (K) 23 4.7%

Met (M) 9 1.8%

Phe (F) 28 5.7%

Pro (P) 50 10.2%

Ser (S) 48 9.8%

Thr (T) 37 7.6%

Trp (W) 1 0.2%

Tyr (Y) 29 5.9%

Val (V) 15 3.1%

Pyl (O) 0 0.0%

Sec (U) 0 0.0%

(B) 0 0.0%

(Z) 0 0.0%

(X) 0 0.0%

Total number of negatively charged residues (Asp + Glu): 19

Total number of positively charged residues (Arg + Lys): 41

**Atomic composition:**

Carbon C 2368

Hydrogen H 3617

Nitrogen N 617

Oxygen O 674

Sulfur S 12

**Formula:** C2368H3617N617O674S12

**Total number of atoms**: 7288

**Extinction coefficients:**

Extinction coefficients are in units of M-1 cm-1, at 280 nm measured in water.

Ext. coefficient 48835

Abs 0.1% (=1 g/l) 0.941, assuming all pairs of Cys residues form cystines

Ext. coefficient 48710

Abs 0.1% (=1 g/l) 0.939, assuming all Cys residues are reduced

**Estimated half-life**:

The N-terminal of the sequence considered is M (Met).

The estimated half-life is: 30 hours (mammalian reticulocytes, in vitro).

>20 hours (yeast, in vivo).

>10 hours (Escherichia coli, in vivo).

**Instability index**:

The instability index (II) is computed to be 26.58

This classifies the protein as stable.

**Aliphatic index:** 71.10

**Grand average of hydropathicity (GRAVY):** -0.130

**SUPPLEMENTARY TABLES**

**Supplementary Table 1:** Physico-chemical properties of the targeted spike glycoprotein sequence of MERS-CoV.

| **Parameter** | **Value** |
| --- | --- |
| Molecular weight | 149505.26 |
| Extinction coefficients Abs. 0.1% (=1 g/l) 1.163, assuming all pairs of Cys residues form cystines | 173845 |
| Ext. coefficient Abs. 0.1% (=1 g/l) 1.145, assuming all Cys residues are reduced | 171220 |
| Theoretical pI | 5.66 |
| Total number of negatively charged residues (Asp + Glu) | 112 |
| Total number of positively charged residues (Arg + Lys) | 95 |
| Half‐Life | 30 hours (mammalian reticulocytes, in vitro).  >20 hours (yeast, in vivo)  >10 hours (Escherichia coli, in vivo). |
| Instability index | 36.53 |
| Grand average of hydropathicity (GRAVY) | -0.064 |
| Aliphatic index | 83.00 |

**Supplementary Table 2:** Predicted CTL epitopes. The epitopes are predicted by using the NetCTL 1.2 server.

| **Supertypes** | **Epitopes** | **Position** | **Prediction score** | **Allergenicity** | **Antigenicity score** |
| --- | --- | --- | --- | --- | --- |
| **A1** | FSSRYVDLY | 266 | 3.4396 | ALLERGEN | 1.0488 |
|  | QVDQLNSTY | 769 | 3.3074 | NON-ALLERGEN | 0.6941 |
|  | NTTLLDLTY | 1256 | 3.0628 | ALLERGEN | 1.5316 |
|  | WSYTGSSFY | 1184 | 2.6591 | NON-ALLERGEN | 1.0625 |
|  | TTITKPLKY | 489 | 2.5338 | NON-ALLERGEN | 0.1377 |
|  | LLDFSVDGY | 324 | 2.5143 | ALLERGEN | 0.0436 |
|  | FSFGVTHEY | 786 | 2.4473 | NON-ALLERGEN | 1.7954 |
|  | FSDGKMGRF | 156 | 2.3162 | ALLERGEN | 1.0567 |
|  | ATDCSDGNY | 211 | 3.5657 | NON-ALLERGEN | 0.7838 |
|  | HISSTMSQY | 681 | 2.1529 | ALLERGEN | 0.8394 |
|  | MTEQLQMGF | 563 | 1.76 | NON-ALLERGEN | 1.2508 |
|  | VVNAPNGLY | 1127 | 1.7566 | ALLERGEN | 0.0211 |
|  | ILEPRSGNY | 186 | 1.6429 | NON-ALLERGEN | 1.1806 |
|  | QTAQGVHLF | 258 | 1.5857 | ALLERGEN | 0.3715 |
|  | RSAIEDLLF | 887 | 1.4916 | NON-ALLERGEN | 0.1712 |
|  | VVKALNESY | 1272 | 0.8317 | ALLERGEN | 0.4901 |
|  | CYSSLIVDY | 437 | 0.8205 | ALLERGEN | 0.3237 |
|  | RCCDRYEEY | 1353 | 0.8184 | ALLERGEN | 0.4485 |
|  | YTSSLLGSI | 947 | 0.7935 | ALLERGEN | 0.303 |
|  | MLKRRDSTY | 696 | 0.8621 | NON-ALLERGEN | 0.908 |
| **A2** | KLQPLTFLL | 317 | 1.4454 | NON-ALLERGEN | 1.1493 |
|  | SLLGSIAGV | 950 | 1.393 | NON-ALLERGEN | 0.2283 |
|  | TLLDLTYEM | 1258 | 1.3851 | ALLERGEN | 1.5684 |
|  | FVYDAYQNL | 630 | 1.3005 | ALLERGEN | 0.0815 |
|  | ALALCVFFI | 1309 | 1.2909 | NON-ALLERGEN | 0.1741 |
|  | SIFYRLNGV | 975 | 1.1492 | ALLERGEN | 0.2263 |
|  | GLVNSSLLV | 716 | 1.1162 | NON-ALLERGEN | 0.3087 |
|  | ALNESYIDL | 1275 | 1.075 | ALLERGEN | 0.9494 |
|  | SIGDIIQRL | 1050 | 1.0623 | ALLERGEN | 0.3824 |
|  | KTWPRPIDV | 42 | 0.8673 | NON-ALLERGEN | 0.4059 |
| **A3** | MLSLQQVVK | 1266 | 1.3878 | NON-ALLERGEN | 0.5668 |
|  | ITYQGLFPY | 69 | 1.3795 | ALLERGEN | 0.9146 |
|  | KLQPLTFLL | 317 | 0.7568 | NON-ALLERGEN | 1.1493 |
|  | MLKRRDSTY | 696 | 0.9329 | NON-ALLERGEN | 0.9068 |
| **A24** | VYKLQPLTF | 315 | 2.0063 | ALLERGEN | 1.1127 |
|  | KLQPLTFLL | 317 | 0.7629 | NON-ALLERGEN | 1 |
| **A26** | ETKTHATLF | 666 | 1.92 | NON-ALLERGEN | 0.2853 |
|  | DAYQNLVGY | 663 | 1.7334 | ALLERGEN | -0.3941 |
|  | DVKQFANGF | 108 | 1.5287 | NON-ALLERGEN | -0.4723 |
|  | TTITKPLKY | 489 | 1.511 | NON-ALLERGEN | 0.1377 |
|  | PVYDTIKYY | 285 | 1.5002 | NON-ALLERGEN | -0.1104 |
|  | FSFGVTHEY | 786 | 1.4849 | NON-ALLERGEN | 1.7954 |
|  | WSYTGSSFY | 1184 | 1.1805 | NON-ALLERGEN | 1.0625 |
|  | NTTLLDLTY | 1256 | 1.0331 | ALLERGEN | 1.5316 |
| **B7** | LVRSESAAL | 1086 | 1.525 | NON-ALLERGEN | 0.4136 |
|  | VASGSTVAM | 555 | 1.4615 | NON-ALLERGEN | 0.5481 |
|  | SPLEGGGWL | 546 | 1.4339 | NON-ALLERGEN | -0.0495 |
|  | YSRSTRSML | 689 | 1.3624 | NON-ALLERGEN | 0.1255 |
|  | YPLSMKSDL | 448 | 1.3329 | ALLERGEN | 0.597 |
|  | SVRSVPGEM | 749 | 1.3278 | ALLERGEN | 0.2918 |
|  | YPSNHIEVV | 1142 | 1.3115 | ALLERGEN | 0.6453 |
|  | WPWYIWLGF | 1295 | 1.2604 | ALLERGEN | 1.4953 |
|  | LPDGCGTLL | 172 | 1.2562 | ALLERGEN | -0.6521 |
|  | AVNNNAQAL | 1025 | 1.2558 | ALLERGEN | 0.3749 |
|  | IASNCYSSL | 433 | 1.2337 | ALLERGEN | 0.455 |
|  | AAANSTGTV | 122 | 1.1783 | ALLERGEN | 0.4499 |
|  | NPTNCIAPV | 1160 | 1.1725 | ALLERGEN | 0.7299 |
| **B8** | MLKRRDSTY | 696 | 1.7688 | NON-ALLERGEN | 0.9068 |
|  | YYRKQLSPL | 540 | 1.7538 | NON-ALLERGEN | 1.3702 |
|  | EMRLASIAF | 756 | 1.646 | ALLERGEN | 1.7706 |
|  | FCSKINQAL | 827 | 1.5315 | ALLERGEN | 0.4218 |
|  | YFNLRNCTF | 231 | 1.5209 | ALLERGEN | 0.7411 |
|  | LLRAFYCIL | 179 | 1.406 | NON-ALLERGEN | -0.3499 |
|  | TIRKIYPAF | 139 | 1.3789 | NON-ALLERGEN | 0.6049 |
|  | YSRSTRSML | 689 | 1.3622 | NON-ALLERGEN | 0.1255 |
|  | MGRFFNHTL | 161 | 1.3148 | ALLERGEN | -0.5312 |
|  | IANKFNQAL | 997 | 1.2711 | NON-ALLERGEN | 0.0036 |
|  | LVRSESAAL | 1086 | 1.2136 | NON-ALLERGEN | 0.4136 |
|  | KLQPLTFLL | 317 | 0.8034 | NON-ALLERGEN | 1.1493 |
| **B27** | RRDSTYGPL | 699 | 1.5815 | NON-ALLERGEN | 1.4262 |
|  | GRLTTLNAF | 1073 | 1.4479 | ALLERGEN | 0.1765 |
|  | SRSTRSMLK | 690 | 1.3306 | ALLERGEN | 0.0105 |
|  | SQFNYKQSF | 465 | 1.2919 | NON-ALLERGEN | 1.1913 |
|  | VRNLFASVK | 846 | 1.1917 | NON-ALLERGEN | 0.441 |
|  | NRNASLNSF | 220 | 1.1182 | NON-ALLERGEN | 0.3572 |
|  | LRACVSVPV | 651 | 1.095 | ALLERGEN | -0.8753 |
|  | TRIVDEWSY | 1178 | 1.0693 | NON-ALLERGEN | -0.0569 |
|  | ARDLICAQY | 920 | 1.0632 | NON-ALLERGEN | 0.9048 |
|  | GQGTHIVSF | 1118 | 1.0349 | ALLERGEN | 0.4695 |
|  | FQFATLPVY | 279 | 1.0284 | NON-ALLERGEN | 1.0243 |
|  | SQLGNCVEY | 598 | 1.0007 | ALLERGEN | 0.2715 |
|  | DRKAWAAFY | 306 | 0.9786 | ALLERGEN | 0.4175 |
|  | GRTYSNITI | 61 | 0.9604 | NON-ALLERGEN | 0.8683 |
| **B39** | RQDDSVRNL | 841 | 1.4981 | ALLERGEN | -0.0516 |
|  | FGGDFNLTL | 865 | 1.4289 | NON-ALLERGEN | 0.7163 |
|  | FVYDAYQNL | 630 | 1.3971 | ALLERGEN | 0.0815 |
|  | SDDGNYYCL | 643 | 1.2606 | ALLERGEN | 0.3011 |
|  | MEAAYTSSL | 943 | 1.1525 | ALLERGEN | 0.5798 |
|  | FSNPTCLIL | 473 | 1.1523 | ALLERGEN | 0.4524 |
|  | YAPEPITSL | 1192 | 1.1315 | NON-ALLERGEN | 0.3549 |
|  | RRDSTYGPL | 699 | 1.1004 | NON-ALLERGEN | 1.4262 |
|  | KETKTHATL | 665 | 1.0589 | NON-ALLERGEN | 0.6779 |
|  | LQTPVGCVL | 707 | 1.0502 | NON-ALLERGEN | -0.1092 |
| **B44** | IEVVSAYGL | 1147 | 1.9044 | ALLERGEN | 0.5933 |
|  | MEAAYTSSL | 943 | 1.8494 | ALLERGEN | 0.5798 |
|  | VECDFSPLL | 381 | 1.6789 | NON-ALLERGEN | 1.3844 |
|  | KETKTHATL | 665 | 1.618 | NON-ALLERGEN | 0.6779 |
|  | SELSNTFGA | 1038 | 0.85 | ALLERGEN | 0.4312 |
| **B58** | LSIPTNFSF | 780 | 2.1112 | NON-ALLERGEN | 1.1278 |
|  | VSIVPSTVW | 527 | 2.0699 | NON-ALLERGEN | 0.5354 |
|  | RSAIEDLLF | 887 | 2.0015 | NON-ALLERGEN | 0.1712 |
|  | YTYYNKWPW | 1289 | 1.8559 | ALLERGEN | 0.725 |
|  | QSSPIIPGF | 857 | 1.8038 | NON-ALLERGEN | 0.8017 |
|  | KAWAAFYVY | 308 | 1.7864 | ALLERGEN | 0.0862 |
|  | FSFGVTHEY | 786 | 1.7176 | NON-ALLERGEN | 1.7954 |
|  | ITYQGLFPY | 69 | 1.665 | ALLERGEN | 0.9146 |
|  | VSVPVSVIY | 655 | 1.5832 | NON-ALLERGEN | 0.0758 |
|  | LGSIAGVGW | 952 | 1.5738 | NON-ALLERGEN | 0.9013 |
|  | ITEDEILEW | 245 | 1.5448 | NON-ALLERGEN | 0.1074 |
|  | LSSFAAIPF | 964 | 1.5432 | NON-ALLERGEN | 0.7545 |
|  | LASELSNTF | 1036 | 1.4926 | ALLERGEN | 0.3396 |
|  | ITITYQGLF | 67 | 1.4775 | ALLERGEN | 1.2982 |
|  | YSSLIVDYF | 438 | 1.3823 | ALLERGEN | -0.0759 |
| **B62** | FSFGVTHEY | 1086 | 1.508 | NON-ALLERGEN | 1.79 |
|  | FQFATLPVY | 279 | 1.4994 | NON-ALLERGEN | 1.0243 |
|  | SQLGNCVEY | 598 | 1.4474 | ALLERGEN | 0.2715 |
|  | MLKRRDSTY | 696 | 1.4024 | NON-ALLERGEN | 0.9068 |
|  | QQRFVYDAY | 627 | 1.3897 | NON-ALLERGEN | 0.2816 |
|  | SLIVDYFSY | 440 | 1.3888 | ALLERGEN | -0.1226 |
|  | FLLTPTESY | 10 | 1.3728 | ALLERGEN | 0.2812 |
|  | EMRLASIAF | 756 | 1.3637 | ALLERGEN | 1.7706 |
|  | LVRSESAAL | 1086 | 0.9772 | NON-ALLERGEN | 0.4136 |
|  | LSIPTNFSF | 780 | 1.3921 | ALLERGEN | 1.1278 |

**Supplementary Table 3:** Predicted HTL epitopes. The epitopes are predicted by using the NetMHC Pan 3.2 server.

| **Position** | **Epitopes** | **Antigenicity** | **Allergenicity** |
| --- | --- | --- | --- |
| 115 | GFVVRIGAAANSTGT | 0.766 | ALLERGEN |
| 135 | STSATIRKIYPAFML | 0.6381 | NON-ALLERGEN |
| 136 | TSATIRKIYPAFMLG | 0.6902 | NON-ALLERGEN |
| 137 | SATIRKIYPAFMLGS | 0.61 | NON-ALLERGEN |
| 141 | RKIYPAFMLGSSVGN | 0.675 | NON-ALLERGEN |
| 289 | TIKYYSIIPHSIRSI | 0.556 | NON-ALLERGEN |
| 290 | IKYYSIIPHSIRSIQ | 0.6153 | NON-ALLERGEN |
| 291 | KYYSIIPHSIRSIQS | 0.4516 | NON-ALLERGEN |
| 311 | AAFYVYKLQPLTFLL | 0.701 | NON-ALLERGEN |
| 312 | AFYVYKLQPLTFLLD | 0.5425 | NON-ALLERGEN |
| 710 | PVGCVLGLVNSSLLV | 0.4244 | NON-ALLERGEN |
| 711 | VGCVLGLVNSSLLVE | 0.5246 | NON-ALLERGEN |
| 742 | PSTLTLRSVRSVPGE | 0.7277 | ALLERGEN |
| 743 | STLTLRSVRSVPGEM | 0.7133 | ALLERGEN |
| 744 | TLTLRSVRSVPGEMR | 0.8070 | ALLERGEN |
| 773 | LNSTYFKLSIPTNFS | 0.4286 | NON-ALLERGEN |
| 774 | NSTYFKLSIPTNFSF | 0.68 | NON-ALLERGEN |
| 775 | STYFKLSIPTNFSFG | 0.8983 | NON-ALLERGEN |
| 776 | TYFKLSIPTNFSFGV | 0.933 | NON-ALLERGEN |
| 777 | YFKLSIPTNFSFGVT | 1.12 | ALLERGEN |
| 845 | SVRNLFASVKSSQSS | 0.5681 | NON-ALLERGEN |
| 846 | VRNLFASVKSSQSSP | 0.4995 | NON-ALLERGEN |
| 974 | QSIFYRLNGVGITQQ | 0.7573 | NON-ALLERGEN |
| 1124 | VSFVVNAPNGLYFMH | 0.4899 | NON-ALLERGEN |
| 4 | SVFLLMFLLTPTESY | 1.1959 | NON-ALLERGEN |
| 5 | VFLLMFLLTPTESYV | 1.2438 | NON-ALLERGEN |
| 6 | FLLMFLLTPTESYVD | 1.0619 | NON-ALLERGEN |
| 7 | LLMFLLTPTESYVDV | 0.7425 | NON-ALLERGEN |
| 8 | LMFLLTPTESYVDVG | 0.4045 | NON-ALLERGEN |
| 80 | DHGDMYVYSAGHATG | 0.5298 | NON-ALLERGEN |
| 81 | HGDMYVYSAGHATGT | 0.9778 | NON-ALLERGEN |
| 82 | GDMYVYSAGHATGTT | 1.085 | NON-ALLERGEN |
| 83 | DMYVYSAGHATGTTP | 1.1956 | ALLERGEN |
| 84 | MYVYSAGHATGTTPQ | 1.2011 | NON-ALLERGEN |
| 96 | TPQKLFVANYSQDVK | 0.1899 | NON-ALLERGEN |
| 97 | PQKLFVANYSQDVKQ | 0.1387 | NON-ALLERGEN |
| 98 | QKLFVANYSQDVKQF | -0.2093 | NON-ALLERGEN |
| 110 | KQFANGFVVRIGAAA | 0.2637 | NON-ALLERGEN |
| 111 | QFANGFVVRIGAAAN | 0.67 | NON-ALLERGEN |
| 112 | FANGFVVRIGAAANS | 0.2453 | NON-ALLERGEN |
| 113 | ANGFVVRIGAAANST | 0.3707 | NON-ALLERGEN |
| 114 | NGFVVRIGAAANSTG | 0.3733 | NON-ALLERGEN |
| 275 | GGNMFQFATLPVYDT | 0.3912 | ALLERGEN |
| 276 | GNMFQFATLPVYDTI | 0.2491 | ALLERGEN |
| 277 | NMFQFATLPVYDTIK | 0.3515 | ALLERGEN |
| 278 | MFQFATLPVYDTIKY | 0.4505 | ALLERGEN |
| 465 | SQFNYKQSFSNPTCL | 0.826 | NON-ALLERGEN |
| 466 | QFNYKQSFSNPTCLI | 0.7237 | NON-ALLERGEN |
| 467 | FNYKQSFSNPTCLIL | 0.8243 | ALLERGEN |
| 474 | SNPTCLILATVPHNL | 0.7347 | ALLERGEN |
| 475 | NPTCLILATVPHNLT | 0.9683 | ALLERGEN |
| 476 | PTCLILATVPHNLTT | 0.5684 | ALLERGEN |
| 477 | TCLILATVPHNLTTI | 0.6471 | ALLERGEN |
| 478 | CLILATVPHNLTTIT | 0.729 | NON-ALLERGEN |
| 548 | LEGGGWLVASGSTVA | 0.1182 | ALLERGEN |
| 549 | EGGGWLVASGSTVAM | 0.1129 | ALLERGEN |
| 550 | GGGWLVASGSTVAMT | 0.2673 | ALLERGEN |
| 551 | GGWLVASGSTVAMTE | 0.4582 | ALLERGEN |
| 552 | GWLVASGSTVAMTEQ | 0.6306 | ALLERGEN |
| 553 | WLVASGSTVAMTEQL | 0.455 | ALLERGEN |
| 563 | MTEQLQMGFGITVQY | 1.3980 | ALLERGEN |
| 564 | TEQLQMGFGITVQYG | 1.4672 | ALLERGEN |
| 565 | EQLQMGFGITVQYGT | 1.3958 | ALLERGEN |
| 645 | DGNYYCLRACVSVPV | -0.1049 | NON-ALLERGEN |
| 646 | GNYYCLRACVSVPVS | 0.0825 | NON-ALLERGEN |
| 647 | NYYCLRACVSVPVSV | 0.327 | NON-ALLERGEN |
| 648 | YYCLRACVSVPVSVI | 0.1058 | NON-ALLERGEN |
| 649 | YCLRACVSVPVSVIY | 0.115 | NON-ALLERGEN |
| 650 | CLRACVSVPVSVIYD | -0.0794 | NON-ALLERGEN |
| 961 | TAGLSSFAAIPFAQS | 0.7120 | NON-ALLERGEN |
| 962 | AGLSSFAAIPFAQSI | 0.6768 | NON-ALLERGEN |
| 963 | GLSSFAAIPFAQSIF | 0.5105 | NON-ALLERGEN |
| 964 | LSSFAAIPFAQSIFY | 0.4313 | NON-ALLERGEN |
| 1070 | LINGRLTTLNAFVAQ | 0.3104 | NON-ALLERGEN |
| 1071 | INGRLTTLNAFVAQQ | 0.3794 | ALLERGEN |
| 1072 | NGRLTTLNAFVAQQL | 0.2653 | ALLERGEN |
| 1073 | GRLTTLNAFVAQQLV | 0.1321 | NON-ALLERGEN |
| 1074 | RLTTLNAFVAQQLVR | 0.0041 | NON-ALLERGEN |
| 1075 | LTTLNAFVAQQLVRS | -0.0018 | NON-ALLERGEN |
| 1076 | TTLNAFVAQQLVRSE | 0.0701 | NON-ALLERGEN |
| 1077 | TLNAFVAQQLVRSES | -0.0064 | NON-ALLERGEN |
| 1078 | LNAFVAQQLVRSESA | -0.0747 | NON-ALLERGEN |
| 1079 | NAFVAQQLVRSESAA | 0.1183 | NON-ALLERGEN |
| 1080 | AFVAQQLVRSESAAL | 0.3883 | NON-ALLERGEN |
| 1081 | FVAQQLVRSESAALS | 0.4314 | NON-ALLERGEN |
| 1082 | VAQQLVRSESAALSA | 0.45 | NON-ALLERGEN |
| 1083 | AQQLVRSESAALSAQ | 0.3358 | NON-ALLERGEN |

**Supplementary Table 4:** Selected helper T-lymphocytes epitope included in the vaccine constructs. The epitopes are divided into strong and weak binders with respective HLA_DRB1 alleles based on the binding score of less than 2% and 10%, respectively.

| **Epitopes (position )** | **MHC class 11 alleles** | **Binding score (%)** | **Remarks** | **Antigenicity** |
| --- | --- | --- | --- | --- |
| TIKYYSIIPHSIRSI (289) | DRB1*01:01 | 0.3 | Strong binder | 0.556 |
|  | DRB1*04:01 | 1.2 | Strong binder |  |
|  | DRB1*07:01 | 0.4 | Strong binder |  |
|  | DRB1*08:01 | 0.6 | Strong binder |  |
|  | DRB1*08:03 | 2.5 | Weak binder |  |
|  | DRB1*10:01 | 0.4 | Strong binder |  |
|  | DRB1*11:01 | 0.8 | Strong binder |  |
|  | DRB1*12:01 | 6.5 | Weak binder |  |
|  | DRB1*13:01 | 3 | Weak binder |  |
|  | DRB1*13:02 | 6 | Weak binder |  |
|  | DRB1*14:01 | 1.9 | Strong binder |  |
|  | DRB1*15:01 | 0.6 | Strong binder |  |
| IKYYSIIPHSIRSIQ (290) | DRB1*01:01 | 0.4 | Strong binder | 0.6153 |
|  | DRB1*04:01 | 1.8 | Strong binder |  |
|  | DRB1*07:01 | 0.5 | Strong binder |  |
|  | DRB1*08:01 | 0.9 | Strong binder |  |
|  | DRB1*08:03 | 3.5 | Weak binder |  |
|  | DRB1*10:01 | 0.4 | Strong binder |  |
|  | DRB1*11:01 | 0.9 | Strong binder |  |
|  | DRB1*12:01 | 7.5 | Weak binder |  |
|  | DRB1*13:01 | 2.5 | Weak binder |  |
|  | DRB1*13:02 | 5 | Weak binder |  |
|  | DRB1*14:01 | 2.5 | Weak binder |  |
|  | DRB1*15:01 | 1.6 | Strong binder |  |
| KYYSIIPHSIRSIQS (291) | DRB1*01:01 | 0.8 | Strong binder | 0.4516 |
|  | DRB1*04:01 | 4 | Weak binder |  |
|  | DRB1*07:01 | 0.4 | Strong binder |  |
|  | DRB1*08:01 | 2.5 | Weak binder |  |
|  | DRB1*08:03 | 5.5 | Weak binder |  |
|  | DRB1*10:01 | 1 | Strong binder |  |
|  | DRB1*11:01 | 1.5 | Strong binder |  |
|  | DRB1*12:01 | 10 | Weak binder |  |
|  | DRB1*13:01 | 2.5 | Weak binder |  |
|  | DRB1*13:02 | 6 | Weak binder |  |
|  | DRB1*14:01 | 4.5 | Weak binder |  |
|  | DRB1*15:01 | 4 | Weak binder |  |
| LNSTYFKLSIPTNFS (773) | DRB1*01:01 | 1 | Strong binder | 0.4286 |
|  | DRB1*04:01 | 0.5 | Strong binder |  |
|  | DRB1*07:01 | 0.4 | Strong binder |  |
|  | DRB1*08:01 | 6.5 | Weak binder |  |
|  | DRB1*08:03 | 6.5 | Weak binder |  |
|  | DRB1*10:01 | 0.3 | Strong binder |  |
|  | DRB1*11:01 | 7 | Weak binder |  |
|  | DRB1*13:02 | 4.5 | Weak binder |  |
|  | DRB1*14:01 | 9.5 | Weak binder |  |
| NSTYFKLSIPTNFSF (774) | DRB1*01:01 | 0.5 | Strong binder | 0.68 |
|  | DRB1*04:01 | 0.25 | Strong binder |  |
|  | DRB1*07:01 | 0.25 | Strong binder |  |
|  | DRB1*08:01 | 4 | Weak binder |  |
|  | DRB1*08:03 | 3.5 | Weak binder |  |
|  | DRB1*10:01 | 0.12 | Strong binder |  |
|  | DRB1*11:01 | 5 | Weak binder |  |
|  | DRB1*13:02 | 3.5 | Weak binder |  |
|  | DRB1*14:01 | 6 | Weak binder |  |
|  | DRB1*15:01 | 7 | Weak binder |  |
| STYFKLSIPTNFSFG (775) | DRB1*01:01 | 0.5 | Strong binder | 0.8983 |
|  | DRB1*04:01 | 0.4 | Strong binder |  |
|  | DRB1*07:01 | 0.3 | Strong binder |  |
|  | DRB1*08:01 | 5.5 | Weak binder |  |
|  | DRB1*08:03 | 4.50 | Weak binder |  |
|  | DRB1*10:01 | 0.15 | Strong binder |  |
|  | DRB1*11:01 | 7.5 | Weak binder |  |
|  | DRB1*13:02 | 4.5 | Weak binder |  |
|  | DRB1*14:01 | 7 | Weak binder |  |
|  | DRB1*15:01 | 7.5 | Weak binder |  |
| TYFKLSIPTNFSFGV (776) | DRB1*01:01 | 0.8 | Strong binder | 0.933 |
|  | DRB1*04:01 | 0.6 | Strong binder |  |
|  | DRB1*07:01 | 0.25 | Strong binder |  |
|  | DRB1*08:01 | 4.5 | Weak binder |  |
|  | DRB1*08:03 | 5.50 | Weak binder |  |
|  | DRB1*10:01 | 0.3 | Strong binder |  |
|  | DRB1*11:01 | 5.5 | Weak binder |  |
|  | DRB1*13:02 | 3.5 | Weak binder |  |
|  | DRB1*14:01 | 8 | Weak binder |  |
|  | DRB1*15:01 | 5.5 | Weak binder |  |
| QSIFYRLNGVGITQQ (974) | DRB1*01:01 | 1 | Strong binder | 0.7573 |
|  | DRB1*04:01 | 5 | Weak binder |  |
|  | DRB1*07:01 | 2.5 | Weak binder |  |
|  | DRB1*10:01 | 2.5 | Weak binder |  |
|  | DRB1*13:02 | 2.5 | Weak binder |  |
|  | DRB1*15:01 | 8.5 | Weak binder |  |
| VSFVVNAPNGLYFMH (1124) | DRB1*01:01 | 1.6 | Strong binder | 0.4899 |
|  | DRB1*04:01 | 3 | Weak binder |  |
|  | DRB1*07:01 | 1.4 | Strong binder |  |
|  | DRB1*08:03 | 0.7 | Strong binder |  |
|  | DRB1*10:01 | 3.5 | Weak binder |  |
|  | DRB1*12:01 | 8.5 | Weak binder |  |
|  | DRB1*13:01 | 0.08 | Strong binder |  |
|  | DRB1*14:01 | 3.5 | Weak binder |  |
|  | DRB1*15:01 | 7 | Weak binder |  |

**Supplementary Table 5:** Overlapping HTL and CTL epitopes sequences.

| **Epitope sequences** | **HLA class I supertypes (Combined score) and alleles (IC50)** | **HLA class II alleles (Binding score%)** |
| --- | --- | --- |
| TIKYYSIIPHSIRSI | A24 (1.9055), B39 (0.9238),  HLA-C*14:02 (14.2), HLA-C*03:03 (5.10), HLA-C*12:03 (8.82), HLA-A*68:01 (24.80), HLA-C*07:02 (43.67), HLA-A*23:01 (43.80) | DRB1*01:01 (0.3), DRB1*04:01 (1.2), DRB1*07:01 (0.4), DRB1*08:01 (0.6),  DRB1*10:01 (0.4), DRB1*11:01 (0.8),  DRB1*14:01 (1.9) |
| IKYYSIIPHSIRSIQ | A24 (1.9077), B7 (0.7686), B39 (0.9259),  HLA-C*14:02 (2.73), HLA-C*03:03 (5.10), HLA-C*12:03 (17.79), HLA-A*68:01 (24.80), HLA-C*07:02 (43.67), HLA-A*23:01 (43.80) | DRB1*01:01 (0.4), DRB1*04:01 (1.8),  DRB1*07:01 (0.5), DRB1*08:01 (0.9), DRB1*10:01(0.4), DRB1*11:01 (0.9),  DRB1*15:01 (1.6) |
| KYYSIIPHSIRSIQS | A24 (1.9036), B7 (0.7750), B39 (0.9219),  HLA-C*14:02 (2.73), HLA-C*12:03 (17.79), HLA-C*03:03 (20.79), HLA-A*68:01 (24.80), HLA-C*07:02 (43.67), HLA-A*23:01 (43.80) | DRB1*01:01 (0.8), DRB1*07:01 (0.4),  DRB1*10:01 (1), DRB1*11:01 (1.5) |
| LNSTYFKLSIPTNFS | B27 (0.8192),  HLA-C*14:02 (2.35), HLA-C*12:03 (7.73), HLA-C*03:03 (13.89), | DRB1*01:01 (1), DRB1*04:01 (0.5),  DRB1*07:01 (0.4), DRB1*10:01 (0.3) |
| NSTYFKLSIPTNFSF | A24 (0.9809), B27 (0.823), B58(2.1162), B62 (1.3972),  HLA-C*14:02 (2.3), HLA-C*12:03 (7.71), HLA-C*03:03 (13.89), HLA-B*58:01 (19.24), HLA-B*15:01 (44.16) | DRB1*01:01 (0.5), DRB1*04:01 (0.25),  DRB1*07:01 (0.25), DRB1*10:01 (0.12) |
| STYFKLSIPTNFSFG | A24 (0.9805), B27 (0.8220), B58 (2.1158), B62 (1.396),  HLA-C*14:02 (2.35), HLA-C*12:03 (7.71), HLA-C*03:03 (13.89), HLA-B*58:01 (19.42), HLA-B*15:01 (44.17) | DRB1*01:01 (0.5), DRB1*04:01 (0.4),  DRB1*07:01 (0.3), DRB1*10:01 (0.15) |
| TYFKLSIPTNFSFGV | A24 (0.977), B7(0.8911), B27(0.7979), B58(2.1127), B62(1.3937),  HLA-C*03:03 (13.89), HLA-B*58:01 (19.42), HLA-B*15:01 (44.17) | DRB1*01:01 (0.8), DRB1*04:01 (0.6),  DRB1*07:01 (0.25), DRB1*10:01 (0.3) |
| QSIFYRLNGVGITQQ | A2(1.1590), A3(0.8279), A24(0.984), A26 (0.8917), B8(0.9173),  HLA-C*12:03 (7.23),HLA-C*14:02(12.07), HLA-C*05:01 (33.49), HLA-C*03:03 (39.14) | DRB1*01:01(1) |
| VSFVVNAPNGLYFMH | A1 (1.7696), A2 (0.7653), A3(1.2596), A26(1.3611), B7(0.8002), B58(0.9574), B62(1.4114),  HLA-C*12:03 (8.61), HLA-C*03:03 (10.33), HLA-C*05:01 (13.89), HLA-B*15:02 (18.33), HLA-A*30:02 (40.59), HLA-A*29:02 (48.61) | DRB1*01:01 (1.6), DRB1*07:01 (1.4)  DRB1*08:03 (0.7), DRB1*13:01 (0.08) |

**Supplementary Table 6:** Selected interferon-gamma epitopes included in the vaccine constructs. The epitope was predicted by using the IFN-epitope server.

| **Epitopes Sequence** | **Result** | **Score** | **Antigenicity** |
| --- | --- | --- | --- |
| GFVVRIGAAANSTGT | Positive | 0.043711578 | 0.766 |
| STSATIRKIYPAFML | Positive | 0.12968554 | 0.6381 |
| TSATIRKIYPAFMLG | Positive | 0.23764004 | 0.6902 |
| SATIRKIYPAFMLGS | Positive | 0.13018903 | 0.61 |
| PSTLTLRSVRSVPGE | Positive | 0.71783679 | 0.7277 |
| LNSTYFKLSIPTNFS | Positive | 2 | 0.4286 |

**Supplementary Table 7:** Assessment of allergenicity and toxicity of selected epitopes included in the vaccine constructs. Allergenicity and toxicity of the epitopes predicted by AllerTOP v.2.0, AllergenFP v.1.0, AllerCatPro v.1.7, and ToxinPred server.

| **Epitopes** | **Allergenicity** | | | **Toxicity** |
| --- | --- | --- | --- | --- |
|  | AllerTOP v.2.0 | AllergenFP v.1.0 | AllerCatPro v.1.7 | ToxinPred |
| ATDCSDGNY | NON-ALLERGEN | NON-ALLERGEN | No evidence | Non-Toxin |
| KLQPLTFLL | NON-ALLERGEN | NON-ALLERGEN | No evidence | Non-Toxin |
| LVRSESAAL | NON-ALLERGEN | NON-ALLERGEN | No evidence | Non-Toxin |
| MLKRRDSTY | NON-ALLERGEN | NON-ALLERGEN | No evidence | Non-Toxin |
| RRDSTYGPL | NON-ALLERGEN | NON-ALLERGEN | No evidence | Non-Toxin |
| LSIPTNFSF | NON-ALLERGEN | NON-ALLERGEN | No evidence | Non-Toxin |
| FSFGVTHEY | NON-ALLERGEN | NON-ALLERGEN | No evidence | Non-Toxin |
| TIKYYSIIPHSIRSI | NON-ALLERGEN | NON-ALLERGEN | No evidence | Non-Toxin |
| LNSTYFKLSIPTNFS | NON-ALLERGEN | ALLERGEN | No evidence | Non-Toxin |
| KYYSIIPHSIRSIQS | NON-ALLERGEN | NON-ALLERGEN | No evidence | Non-Toxin |
| IKYYSIIPHSIRSIQ | NON-ALLERGEN | NON-ALLERGEN | No evidence | Non-Toxin |
| NSTYFKLSIPTNFSF | NON-ALLERGEN | ALLERGEN | No evidence | Non-Toxin |
| QSIFYRLNGVGITQQ | NON-ALLERGEN | ALLERGEN | No evidence | Non-Toxin |
| TYFKLSIPTNFSFGV | NON-ALLERGEN | NON-ALLERGEN | No evidence | Non-Toxin |
| VSFVVNAPNGLYFMH | NON-ALLERGEN | ALLERGEN | No evidence | Non-Toxin |
| STYFKLSIPTNFSFG | NON-ALLERGEN | NON-ALLERGEN | No evidence | Non-Toxin |
| GFVVRIGAAANSTGT | NON-ALLERGEN | NON-ALLERGEN | No evidence | Non-Toxin |
| STSATIRKIYPAFML | NON-ALLERGEN | NON-ALLERGEN | No evidence | Non-Toxin |
| TSATIRKIYPAFMLG | NON-ALLERGEN | NON-ALLERGEN | No evidence | Non-Toxin |
| SATIRKIYPAFMLGS | NON-ALLERGEN | NON-ALLERGEN | No evidence | Non-Toxin |
| PSTLTLRSVRSVPGE | NON-ALLERGEN | NON-ALLERGEN | No evidence | Non-Toxin |

**Supplementary Table 8:** Refined the best vaccine structures. The refinement was performed by using Galaxy refine web server.

| **Model** | **GDT-HA** | **RMSD** | **MolProbity** | **Clash score** | **Poor rotamers** | **Rama favored** |
| --- | --- | --- | --- | --- | --- | --- |
| Initial | 1.0000 | 0.000 | 3.501 | 80.8 | 5.9 | 90.3 |
| MODEL 1 | 0.9059 | 0.519 | 2.725 | 34.1 | 2.1 | 92.4 |
| MODEL 2 | 0.9013 | 0.514 | 2.506 | 35.1 | 0.8 | 92.2 |
| MODEL 3 | 0.9013 | 0.514 | 2.628 | 36.0 | 1.3 | 91.4 |
| MODEL 4 | 0.9054 | 0.503 | 2.490 | 33.1 | 1.0 | 92.2 |
| MODEL 5 | 0.9039 | 0.519 | 2.608 | 36.2 | 1.3 | 92.0 |

**Supplementary Table 9:** Post translational modification analysis of the finalized multi-epitopic vaccine construct.

| **Specific post-translational modification** | | | |
| --- | --- | --- | --- |
|  | **Prediction of specific function** | **Server** | **Result** |
| Lipid post-translational modifications (PMTs) | GPI modification site prediction | big-PI/GPI animals | None potential GPI-modification site was found. |
|  | Myristoyl | MyrPS/NMT | 'NO' myristoylation site was found |
| Phosphorylation | Identification of General phosphorylation site | NetPhos | 45 site predicted ( Ser: 24, Thr: 16, Tyr: 5) |
| Glycosylation | N-glycosylation sites | NetNGlyc | No significant N-glycosylation sites found |
|  | O_GlcNAc attachment sites | YinOYang | Position: 388 |
| Acetylation | N-terminal acetylation | NetAct | No significant N-terminal acetylation sites found |

**Supplementary Table 10:** Population coverage selected CTL and HTL epitope in the vaccine construct.

| **Epitope Category** |  | **Population/area** | **Class I** | | |
| --- | --- | --- | --- | --- | --- |
|  |  |  | **Coverage^a^** | **Average_hit^b^** | **pc90^C^** |
| **CTL Epitopes** |  | [Central Africa](http://tools.iedb.org/population/result/#Central%20Africa) | 98.35% | 19.9 | 12.48 |
|  |  | [Central America](http://tools.iedb.org/population/result/#Central%20America) | 9.07% | 0.66 | 0.77 |
|  |  | [East Africa](http://tools.iedb.org/population/result/#East%20Africa) | 98.29% | 19.91 | 12.43 |
|  |  | [East Asia](http://tools.iedb.org/population/result/#East%20Asia) | 100.0% | 33.32 | 24.9 |
|  |  | [Europe](http://tools.iedb.org/population/result/#Europe) | 99.99% | 36.41 | 28.62 |
|  |  | [North Africa](http://tools.iedb.org/population/result/#North%20Africa) | 99.27% | 22.4 | 15.05 |
|  |  | [North America](http://tools.iedb.org/population/result/#North%20America) | 100.0% | 35.14 | 26.89 |
|  |  | [Northeast Asia](http://tools.iedb.org/population/result/#Northeast%20Asia) | 98.0% | 18.98 | 11.54 |
|  |  | [Oceania](http://tools.iedb.org/population/result/#Oceania) | 98.69% | 19.58 | 12.71 |
|  |  | [South Africa](http://tools.iedb.org/population/result/#South%20Africa) | 99.67% | 24.02 | 16.56 |
|  |  | [South America](http://tools.iedb.org/population/result/#South%20America) | 100.0% | 27.73 | 19.64 |
|  |  | [South Asia](http://tools.iedb.org/population/result/#South%20Asia) | 100.0% | 30.12 | 22.06 |
|  |  | [Southeast Asia](http://tools.iedb.org/population/result/#Southeast%20Asia) | 100.0% | 30.05 | 22.1 |
|  |  | [Southwest Asia](http://tools.iedb.org/population/result/#Southwest%20Asia) | 97.66% | 18.87 | 11.11 |
|  |  | [West Africa](http://tools.iedb.org/population/result/#West%20Africa) | 98.88% | 21.13 | 14.2 |
|  |  | [West Indies](http://tools.iedb.org/population/result/#West%20Indies) | 98.64% | 17.93 | 12.34 |
|  |  | [World](http://tools.iedb.org/population/result/#World) | 100.0% | 34.6 | 26.08 |
|  |  | **Average** | **93.91** | **24.16** | **17.03** |
|  |  | **Standard deviation** | **21.23** | **8.62** | **7.1** |
| **Epitope Category** | **Population/area** | | **Class II** | | |
|  |  |  | **Coverage^a^** | **Average_hit^b^** | **pc90^c^** |
| **HTL Epitopes** | [Central Africa](http://tools.iedb.org/population/result/#Central%20Africa) | | 58.94% | 6.3 | 2.19 |
|  | [Central America](http://tools.iedb.org/population/result/#Central%20America) | | 27.48% | 2.63 | 1.24 |
|  | [East Africa](http://tools.iedb.org/population/result/#East%20Africa) | | 63.4% | 6.82 | 2.46 |
|  | [East Asia](http://tools.iedb.org/population/result/#East%20Asia) | | 62.47% | 6.82 | 2.4 |
|  | [Europe](http://tools.iedb.org/population/result/#Europe) | | 87.85% | 11.22 | 7.41 |
|  | [North Africa](http://tools.iedb.org/population/result/#North%20Africa) | | 73.48% | 8.36 | 3.39 |
|  | [North America](http://tools.iedb.org/population/result/#North%20America) | | 86.84% | 10.98 | 6.84 |
|  | [Northeast Asia](http://tools.iedb.org/population/result/#Northeast%20Asia) | | 57.12% | 6.08 | 2.1 |
|  | [Oceania](http://tools.iedb.org/population/result/#Oceania) | | 63.42% | 6.84 | 2.46 |
|  | [South Africa](http://tools.iedb.org/population/result/#South%20Africa) | | 26.21% | 2.37 | 1.22 |
|  | [South America](http://tools.iedb.org/population/result/#South%20America) | | 39.17% | 3.9 | 1.48 |
|  | [South Asia](http://tools.iedb.org/population/result/#South%20Asia) | | 84.45% | 10.44 | 5.79 |
|  | [Southeast Asia](http://tools.iedb.org/population/result/#Southeast%20Asia) | | 57.93% | 6.18 | 2.14 |
|  | [Southwest Asia](http://tools.iedb.org/population/result/#Southwest%20Asia) | | 45.27% | 4.6 | 1.64 |
|  | [West Africa](http://tools.iedb.org/population/result/#West%20Africa) | | 62.15% | 6.71 | 2.38 |
|  | [West Indies](http://tools.iedb.org/population/result/#West%20Indies) | | 66.52% | 7.36 | 2.69 |
|  | [World](http://tools.iedb.org/population/result/#World) | | 80.8% | 9.76 | 4.69 |
|  | **Average** | | **61.38** | **6.9** | **3.09** |
|  | **Standard deviation** | | **18.18** | **2.58** | **1.86** |

**Supplementary Table 11:** Refinement of the best-docked complexes (TLR4-vaccine) on FireDock.

| **Rank** | **Solution Number** | **Global Energy** | **Attractive VdW** | **Repulsive VdW** | **ACE** | **HB** |
| --- | --- | --- | --- | --- | --- | --- |
| 1 | 10 | -21.16 | -42.23 | 38.54 | 9.29 | -6.82 |
| 2 | 7 | -12.11 | -30.60 | 25.48 | 7.16 | -1.40 |
| 3 | 1 | 3.67 | -0.78 | 0.02 | 0.41 | 0.00 |
| 4 | 4 | 11.05 | -7.50 | 1.30 | 3.78 | 0.00 |
| 5 | 3 | 29.61 | -36.49 | 21.50 | 10.85 | -2.81 |
| 6 | 2 | 140.05 | -59.29 | 260.30 | 11.15 | -7.26 |
| 7 | 5 | 335.96 | -53.01 | 503.52 | 8.05 | -5.90 |
| 8 | 6 | 558.83 | -47.11 | 774.34 | 11.72 | -5.84 |
| 9 | 9 | 1589.18 | -22.44 | 1994.18 | 5.40 | -2.08 |
| 10 | 8 | 1678.01 | -44.66 | 2199.01 | 2.69 | -5.91 |

VdW (van der Waals), ACE (Atomic Contact Energy), HB (Hydrogen Bonds Energy)

**Supplementary Table 12:** Refinement of the best-docked complexes (TLR2-vaccine) on FireDock.

| **Rank** | [**Solution Number**](http://bioinfo3d.cs.tau.ac.il/FireDock/bin/showRes.pl?id=TLR2prepared.pdb_MERSCoVvaccine.pdb_50_59_7_8_8_120&from=1&to=20&sortBy=1) | **Global Energy** | [**Attractive VdW**](http://bioinfo3d.cs.tau.ac.il/FireDock/bin/showRes.pl?id=TLR2prepared.pdb_MERSCoVvaccine.pdb_50_59_7_8_8_120&from=1&to=20&sortBy=3) | [**Repulsive VdW**](http://bioinfo3d.cs.tau.ac.il/FireDock/bin/showRes.pl?id=TLR2prepared.pdb_MERSCoVvaccine.pdb_50_59_7_8_8_120&from=1&to=20&sortBy=4) | [**ACE**](http://bioinfo3d.cs.tau.ac.il/FireDock/bin/showRes.pl?id=TLR2prepared.pdb_MERSCoVvaccine.pdb_50_59_7_8_8_120&from=1&to=20&sortBy=5) | [**HB**](http://bioinfo3d.cs.tau.ac.il/FireDock/bin/showRes.pl?id=TLR2prepared.pdb_MERSCoVvaccine.pdb_50_59_7_8_8_120&from=1&to=20&sortBy=6) |
| --- | --- | --- | --- | --- | --- | --- |
| 1 | 10 | -22.31 | -34.45 | 26.29 | 23.41 | -6.78 |
| 2 | 2 | -0.00 | -22.45 | 7.97 | 10.05 | -0.67 |
| 3 | 3 | 4.73 | -14.00 | 7.84 | 5.86 | -1.04 |
| 4 | 9 | 4.77 | -10.36 | 2.87 | 3.59 | -1.58 |
| 5 | 7 | 12.01 | -6.00 | 2.86 | 6.56 | 0.00 |
| 6 | 1 | 24.13 | -36.81 | 7.13 | 20.92 | -4.62 |
| 7 | 4 | 42.39 | -28.71 | 17.55 | 22.52 | -0.51 |
| 8 | 5 | 1359.18 | -66.08 | 1748.50 | 20.21 | -10.00 |
| 9 | 8 | 1923.01 | -82.43 | 2579.13 | -2.53 | -6.94 |
| 10 | 6 | 4761.46 | -60.24 | 6039.18 | 20.78 | -10.90 |

VdW (van der Waals), ACE (Atomic Contact Energy), HB (Hydrogen Bonds Energy)

**Supplementary Table 13:** Molecular docking analysis of selected CTL epitopes included in the vaccine construct with commonly occurring HLA class I allele HLA-A*02:01. Energy scores of the top epitopes-allele docked complexes are displayed in the table.

| **Sl. no** | **CTL Epitopes/control ligand** | **Representative** | **Weighted Score (HLA-A*02:01)** |
| --- | --- | --- | --- |
|  | **Positive control** | **Center** | **-538.2** |
|  |  | **Lowest Energy** | **-562.8** |
|  | **Negative control** | **Center** | **696.2** |
|  |  | **Lowest Energy** | **756.5** |
| 1 | ATDCSDGNY | Center | -486.4 |
|  |  | Lowest Energy | -598.1 |
| 2 | KLQPLTFLL | Center | -630.2 |
|  |  | Lowest Energy | -795.6 |
| 3 | LVRSESAAL | Center | -531.9 |
|  |  | Lowest Energy | -632.1 |
| 4 | MLKRRDSTY | Center | -571.9 |
|  |  | Lowest Energy | -646.0 |
| 5 | RRDSTYGPL | Center | -529.1 |
|  |  | Lowest Energy | -655.1 |
| 6 | LSIPTNFSF | Center | -667.5 |
|  |  | Lowest Energy | -763.6 |
| **7** | FSFGVTHEY | Center | -638.5 |
|  |  | Lowest Energy | -736.5 |

**Supplementary Table 14:** Molecular docking analysis of selected HTL epitopes included in the vaccine construct with commonly occurring HLA class II alleles HLA-DRB1*01:01 and HLA-DRB1*15:01. Energy scores of the top epitopes-allele docked complexes are displayed in the table.

| **Sl. no** | **HTL Epitopes/control ligand** | **Representative** | **Weighted Score (HLA-DRB1*01:01 )** | **Weighted Score (HLA-DRB1*15:01)** |
| --- | --- | --- | --- | --- |
|  | **Positive control** | **Center** | **-597.8** | **-726.5** |
|  |  | **Lowest Energy** | **-638.9** | **-731.0** |
|  | **Negative control** | **Center** | **599.5** | **726.5** |
|  |  | **Lowest Energy** | **725.9** | **794.0** |
| 1 | TIKYYSIIPHSIRSI | Center | -665.4 | -696.2 |
|  |  | Lowest Energy | -766.8 | -756.5 |
| 2 | LNSTYFKLSIPTNFS | Center | -731.0 | -782.8 |
|  |  | Lowest Energy | -929.3 | -975.8 |
| 3 | KYYSIIPHSIRSIQS | Center | -599.5 | -628.1 |
|  |  | Lowest Energy | -725.9 | -752.5 |
| 4 | IKYYSIIPHSIRSIQ | Center | -726.5 | -651.5 |
|  |  | Lowest Energy | -745.9 | -751.2 |
| 5 | NSTYFKLSIPTNFSF | Center | -836.7 | -821.8 |
|  |  | Lowest Energy | -987.0 | -1125.6 |
| 6 | QSIFYRLNGVGITQQ | Center | -823.6 | -817.4 |
|  |  | Lowest Energy | -912.7 | -896.0 |
| 7 | TYFKLSIPTNFSFGV | Center | -726.5 | -759.9 |
|  |  | Lowest Energy | -794.0 | -813.4 |
| 8 | VSFVVNAPNGLYFMH | Center | -867.8 | -883.4 |
|  |  | Lowest Energy | -1046.2 | -960.6 |
| 9 | STYFKLSIPTNFSFG | Center | -763.9 | -795.1 |
|  |  | Lowest Energy | -936.8 | -956.7 |

**SUPPLEMENTARY FIGURE**

**Supplementary Figure S1:** The ERRAT plot of the finalized multi-epitopic vaccine structure.

**
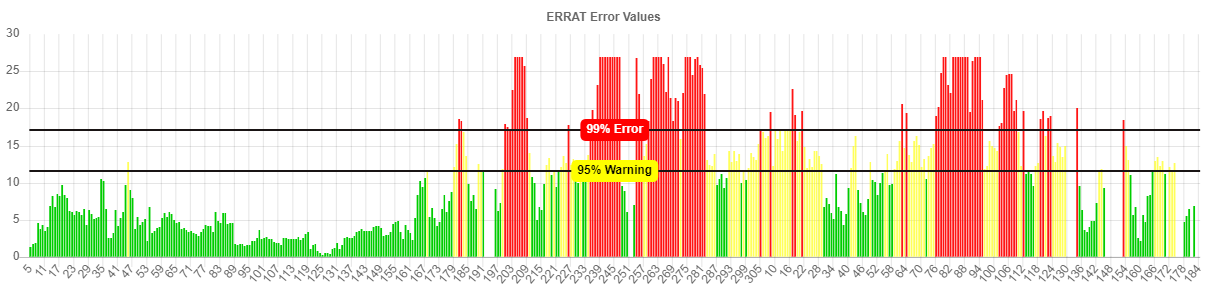
**

**Supplementary Figure S2:** Allergenicity assessment of the final multi-epitope vaccine. The vaccine was found to be non-allergen by (**A**) AllerTOP v2.0 and (**B**) Allergen FP v1.0 sever.

**
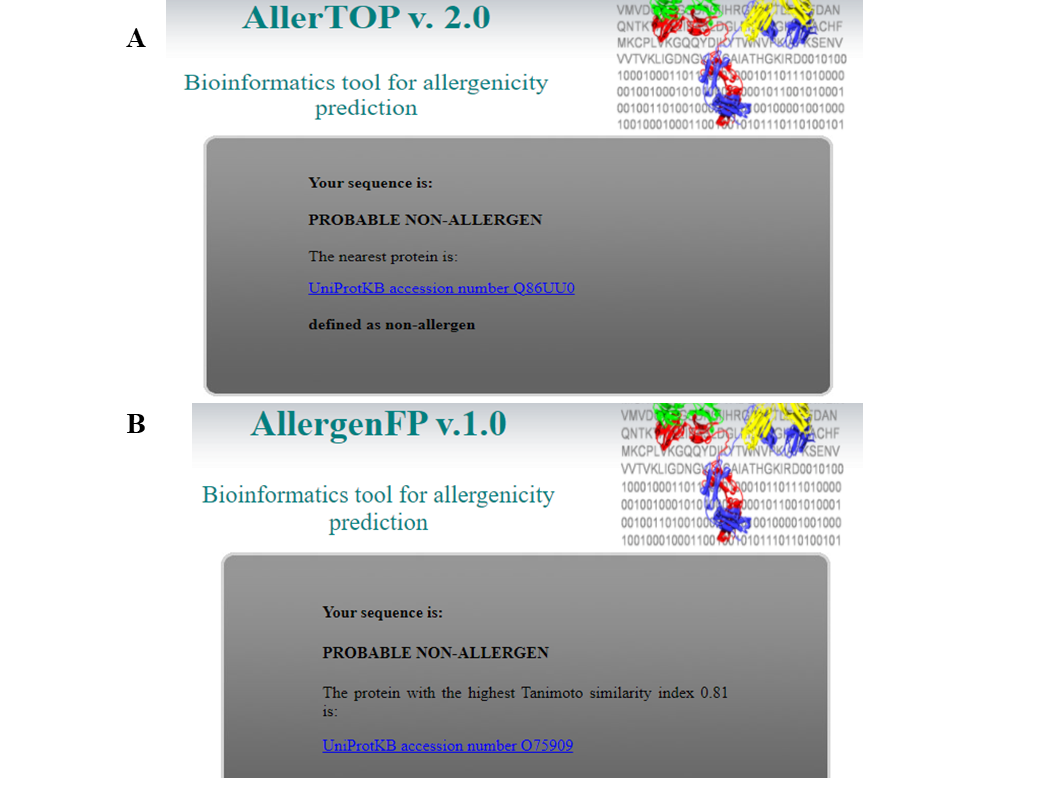
**

**Supplementary Figure S3:** Signal peptide prediction in the vaccine constructs.

Measure Position Value

max. C 19 0.107

max. Y 70 0.106

max. S 60 0.111

mean S 1-69 0.097

D 1-69 0.101 0.450 NO

Name=MERS_CoV vsccine SP='NO' D=0.101 D-cutoff=0.450 Networks=SignalP-noTM

**
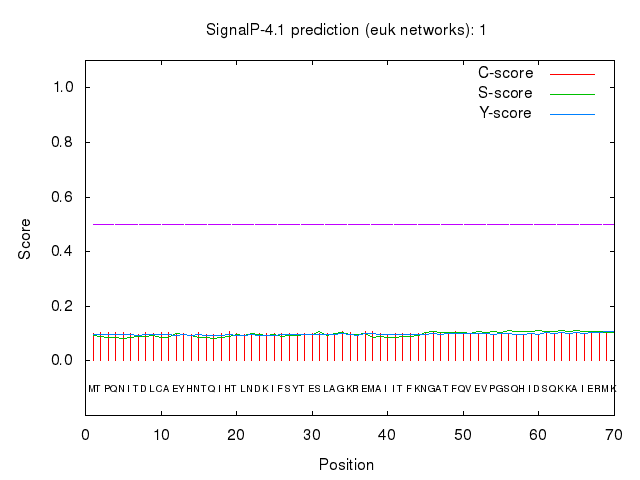
**

**Supplementary Figure S4:** Transmembrane helix prediction in the vaccine constructs.

Length: 489

# Number of predicted TMHs: 0

# Exp number of AAs in TMHs: 6.19638

# Exp number, first 60 AAs: 0.00042

# Total prob of N-in: 0.13512

TMHMM2.0 outside 1 489

**
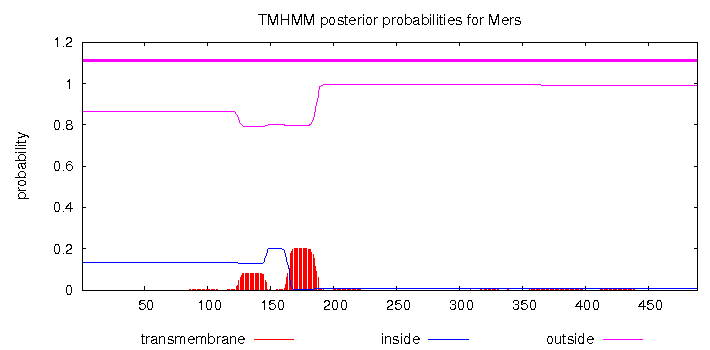
**

**Supplementary Figure S5:** Prediction of N-glycosylation sites in the target protein.

**
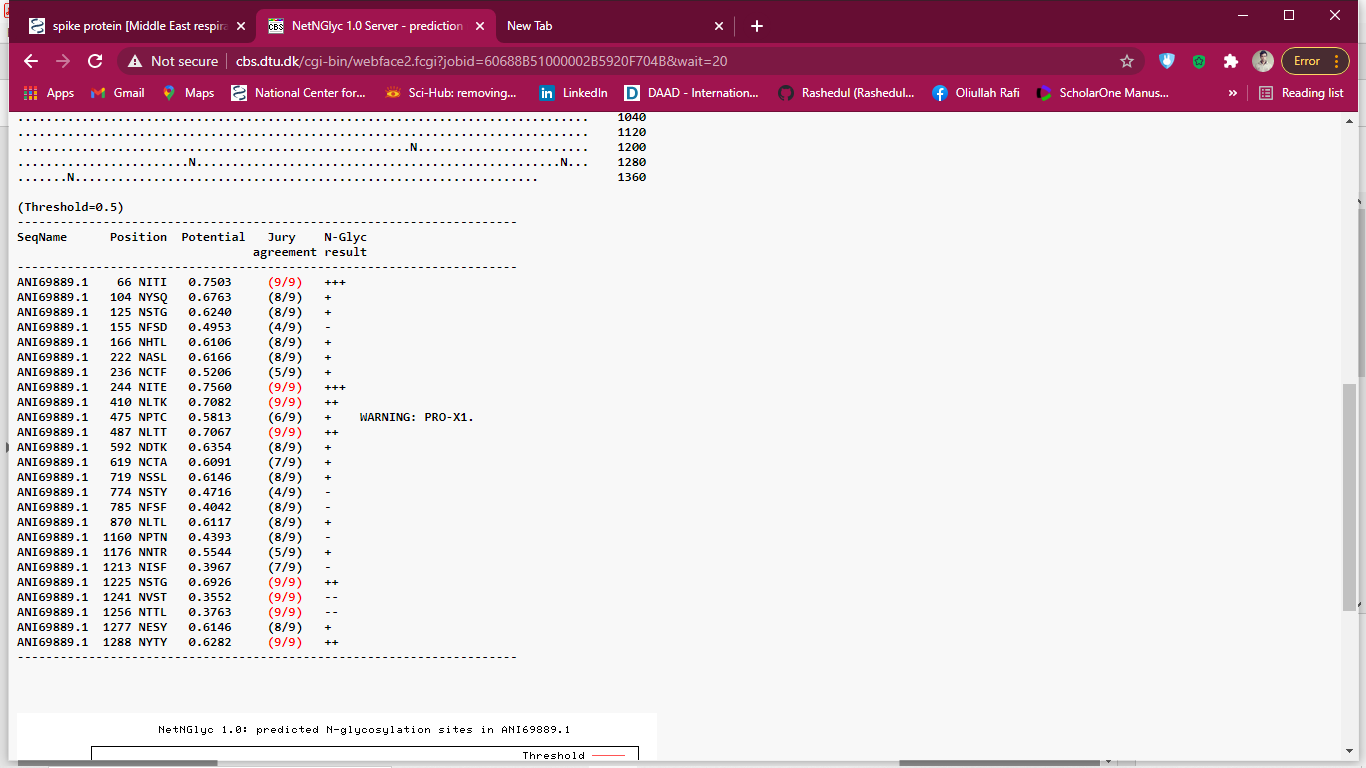
**

**
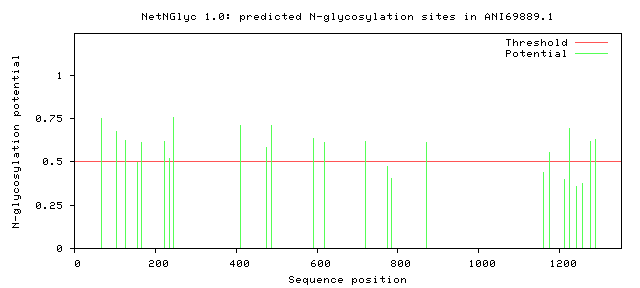
**

**Supplementary Figure S6:** Prediction of O-GlcNAc sites sites in the target protein.

**
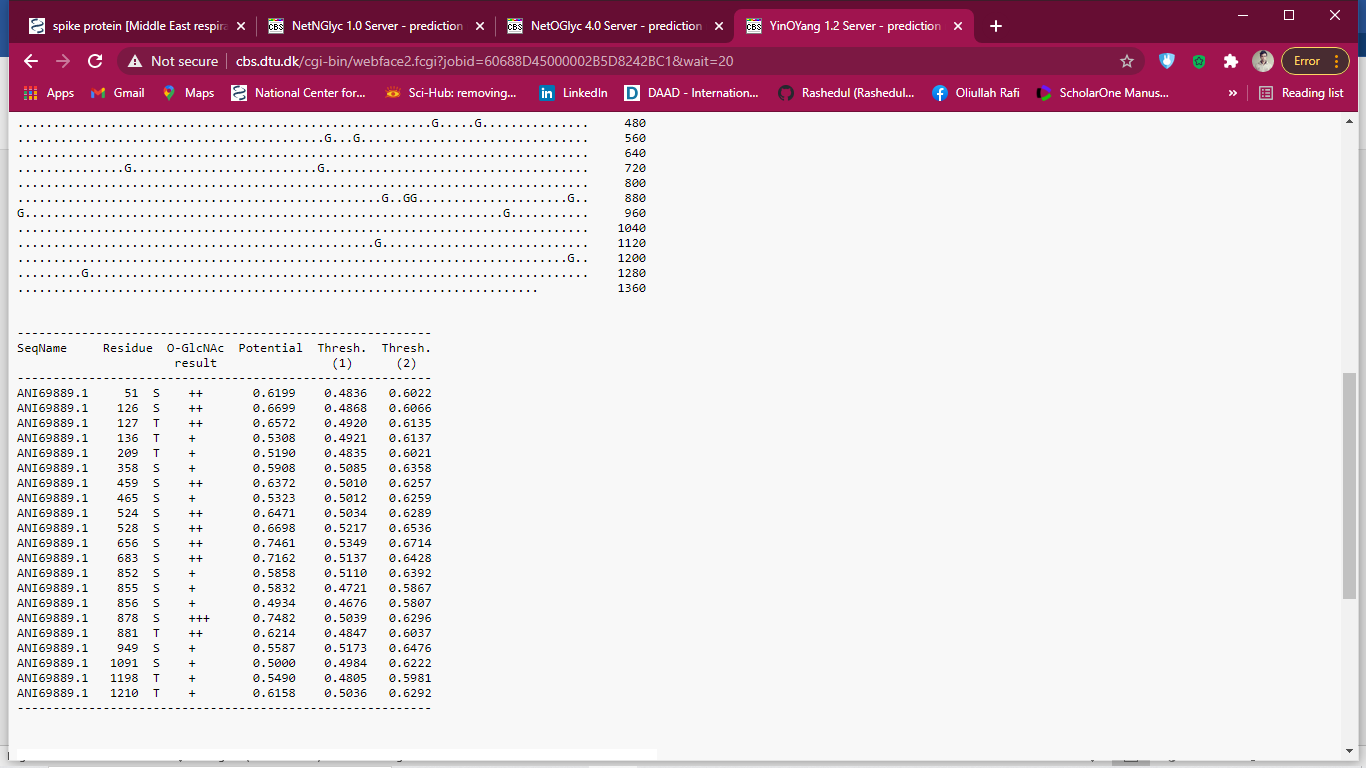
**

**
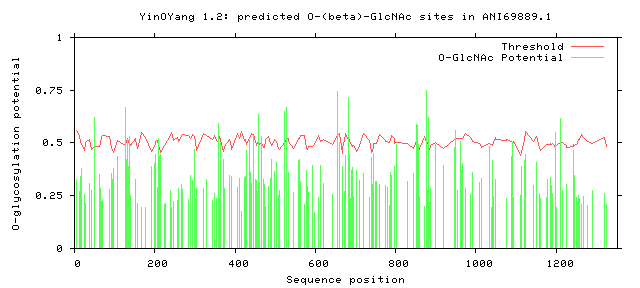
**

**Supplementary Figure S7:** B-cell epitopes.

Continuous epitopes, predicted by ElliPro and visualized using PyMol. Epitopes are shown in green color and the vaccine is shown in hot red color.

**
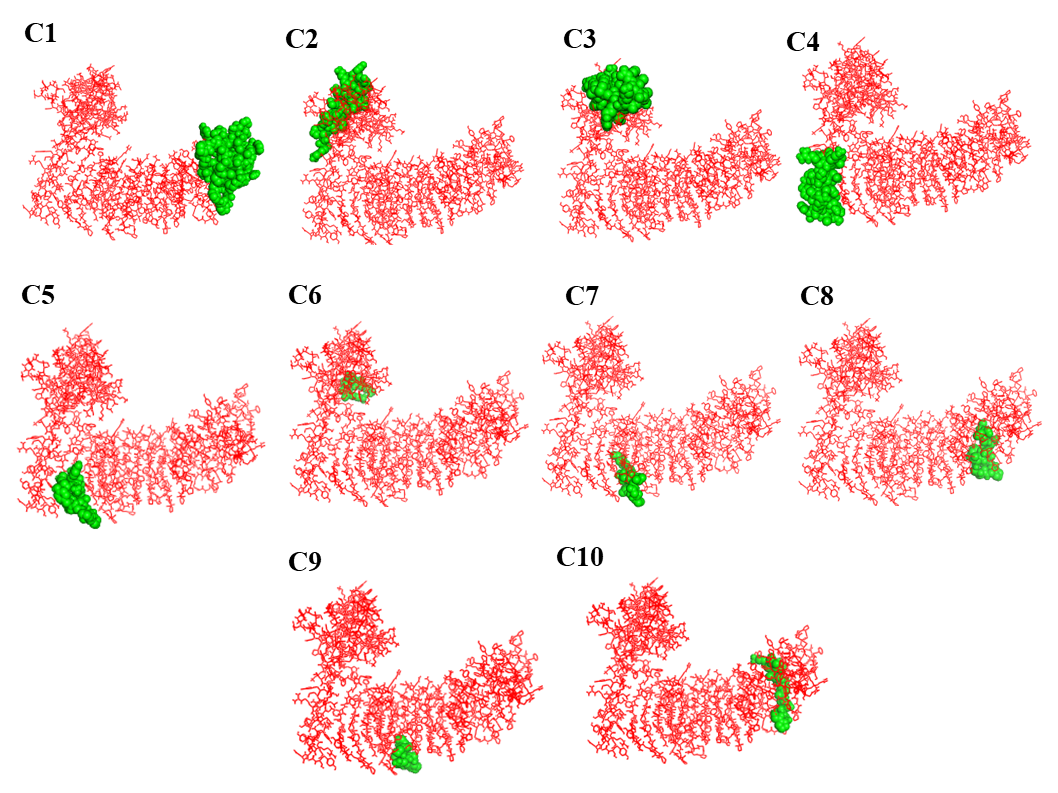
**

**Supplementary Figure S8:** B-cell epitopes.

Discontinuous epitopes predicted by ElliPro and visualized using PyMol. Epitopes are shown in blue color and the vaccine is shown in red color.

**
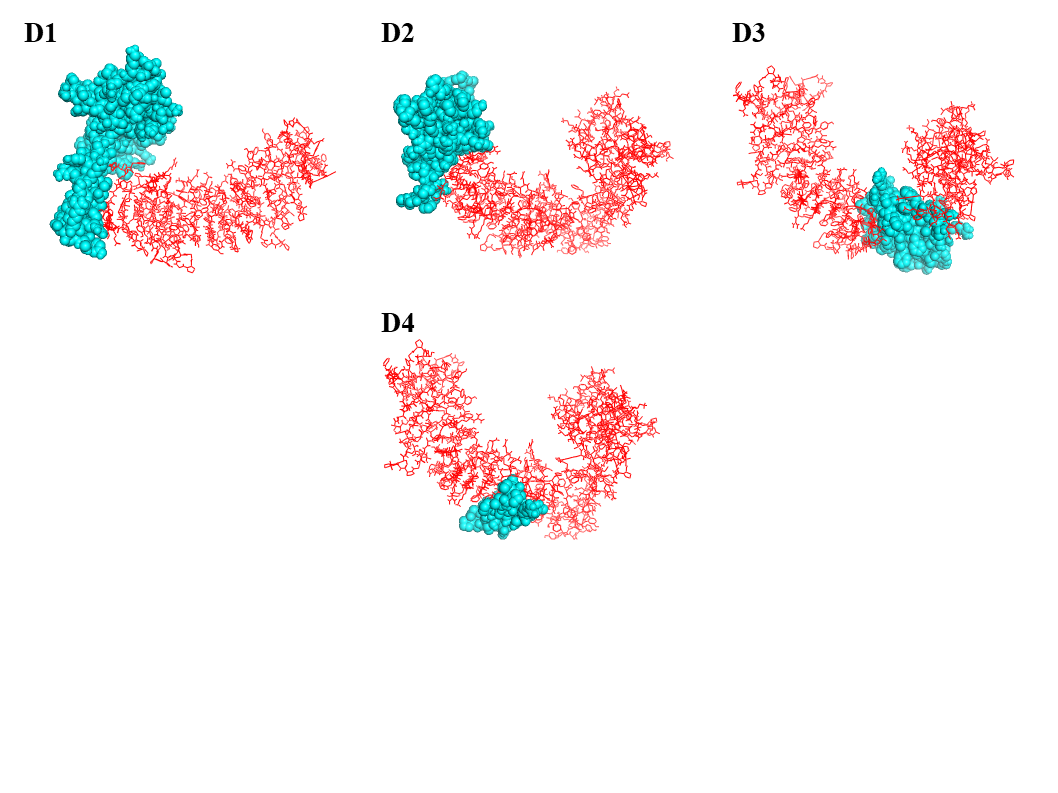
**

**Supplementary Figure S9:** The secondary structure content analysis from (A) TLR-4 and vaccine complex, (B) TLR-2 and vaccine complex.

**
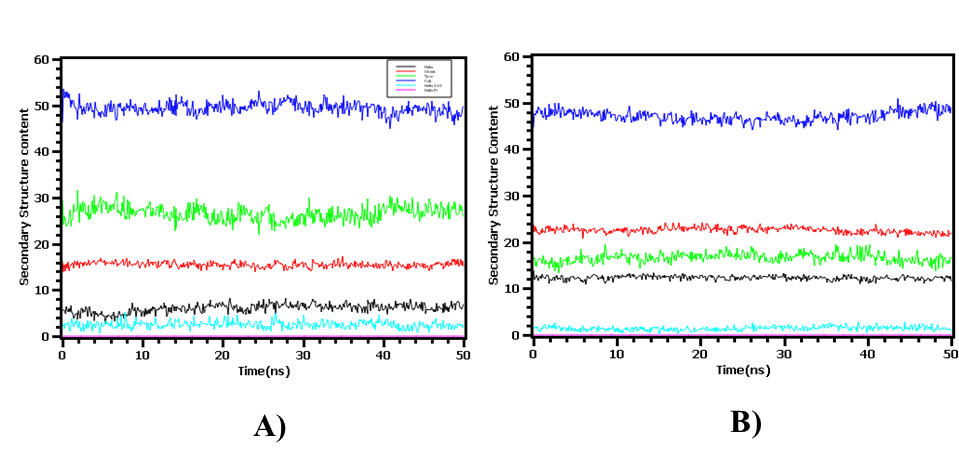
**

**Supplementary Figure S10:** Graphs for immune simulation.


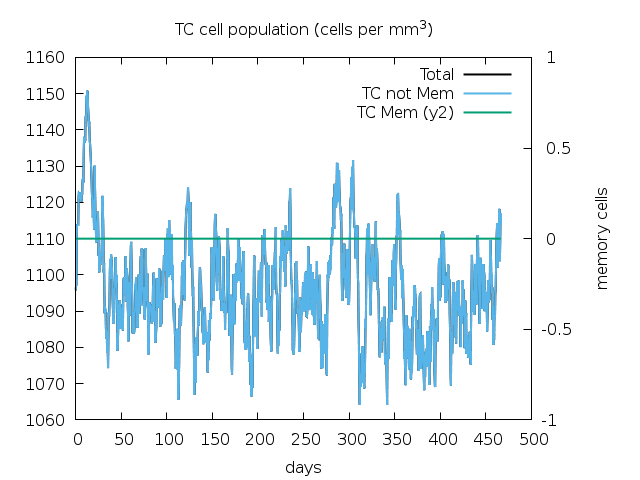

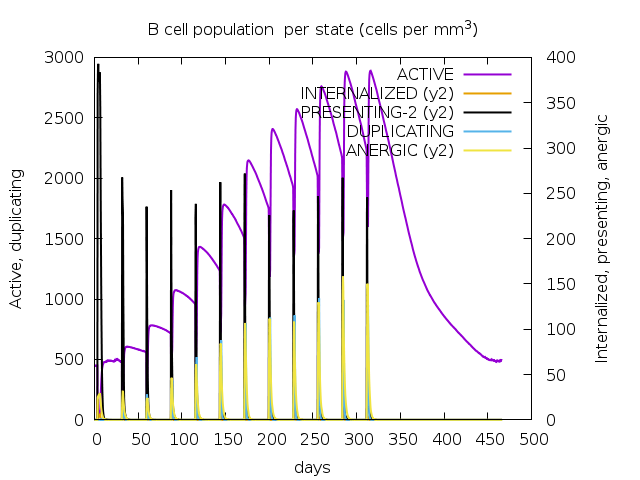

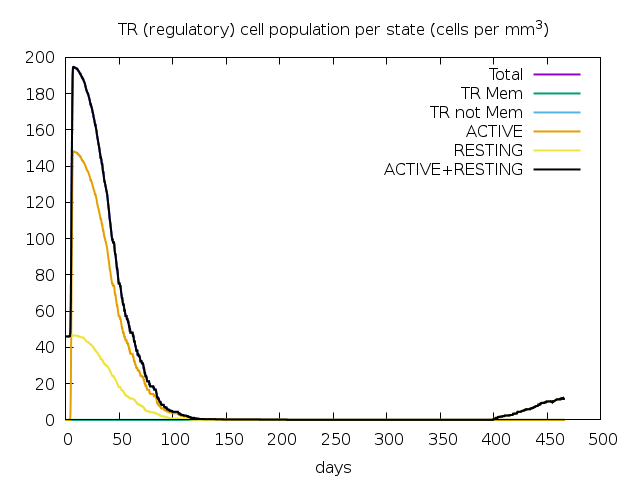

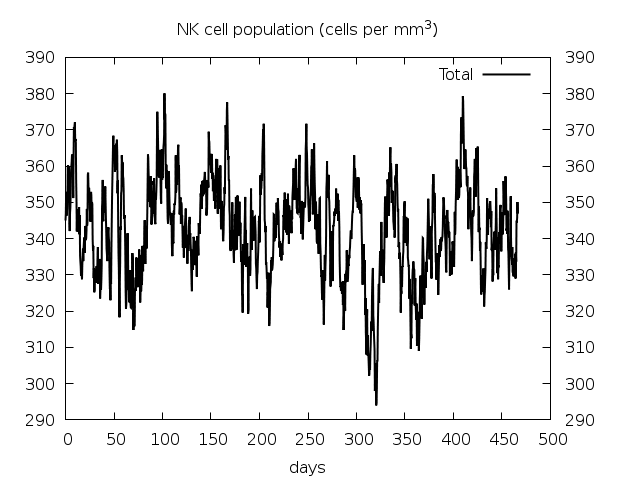

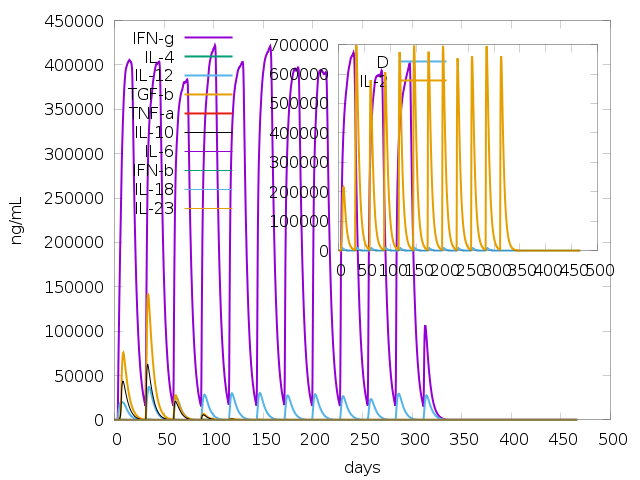

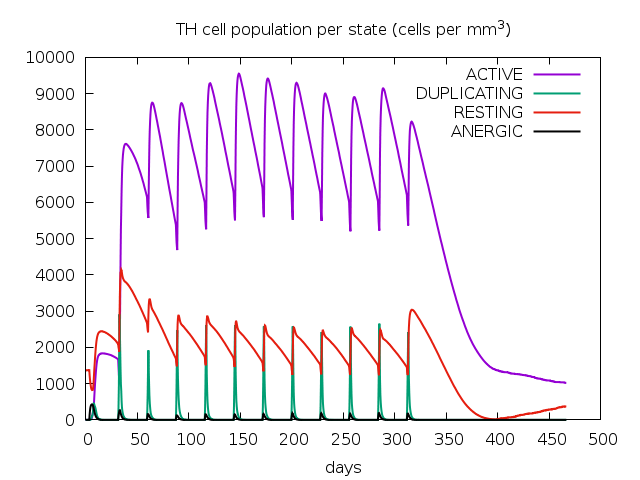


**A**

**B**

**C**

**D**

**E**

**F**
